# Systematic review and meta-analysis of augmentation and combination treatments for early-stage treatment-resistant depression

**DOI:** 10.1177/02698811221104058

**Published:** 2022-07-21

**Authors:** Fraser Scott, Elliot Hampsey, Sam Gnanapragasam, Ben Carter, Lindsey Marwood, Rachael W Taylor, Cansu Emre, Lora Korotkova, Jonatan Martín-Dombrowski, Anthony J Cleare, Allan H Young, Rebecca Strawbridge

**Affiliations:** 1 South London and Maudsley NHS Foundation Trust, London, UK; 2 Department of Psychological Medicine, Institute of Psychiatry, Psychology and Neuroscience, King’s College London, London, UK; 3 Department of Biostatistics and Health Informatics, Institute of Psychiatry, Psychology and Neuroscience, King’s College London, London, UK

**Keywords:** Major depressive disorder, treatment resistant depression, meta-analysis, augmentation, combination

## Abstract

**Background:**

Major depressive disorder (MDD) is a highly burdensome health condition, for which there are numerous accepted pharmacological and psychological interventions. Adjunctive treatment (augmentation/combination) is recommended for the ~50% of MDD patients who do not adequately respond to first-line treatment. We aimed to evaluate the current evidence for concomitant approaches for people with early-stage treatment-resistant depression (TRD; defined below).

**Methods:**

We systematically searched Medline and Institute for Scientific Information Web of Science to identify randomised controlled trials of adjunctive treatment of ⩾10 adults with MDD who had not responded to ⩾1 adequate antidepressant. The cochrane risk of bias (RoB) tool was used to assess study quality. Pre-post treatment meta-analyses were performed, allowing for comparison across heterogeneous study designs independent of comparator interventions.

**Results:**

In total, 115 trials investigating 48 treatments were synthesised. The mean intervention duration was 9 weeks (range 5 days to 18 months) with most studies assessed to have low (*n* = 57) or moderate (*n* = 51) RoB. The highest effect sizes (ESs) were from cognitive behavioural therapy (ES = 1.58, 95% confidence interval (CI): 1.09–2.07), (es)ketamine (ES = 1.48, 95% CI: 1.23–1.73) and risperidone (ES = 1.42, 95% CI: 1.29–1.61). Only aripiprazole and lithium were examined in ⩾10 studies. Pill placebo (ES = 0.89, 95% CI: 0.81–0.98) had a not inconsiderable ES, and only six treatments’ 95% CIs did not overlap with pill placebo’s (aripiprazole, (es)ketamine, mirtazapine, olanzapine, quetiapine and risperidone). We report marked heterogeneity between studies for almost all analyses.

**Conclusions:**

Our findings support cautious optimism for several augmentation strategies; although considering the high prevalence of TRD, evidence remains inadequate for each treatment option.

## Introduction

Major Depressive Disorder (MDD) is one of the most common neuropsychiatric conditions, with an estimated lifetime prevalence of approximately 12% (WHO World Mental Health Survey Consortium, 2004). MDD imposes a substantial burden of illness and is the leading cause of disability internationally (WHO, 2017). The most common treatments for MDD are broadly classified as pharmacological or psychological, with a multitude of different treatments from each category available (Cleare et al., 2015).

A significant proportion of patients with MDD do not respond to treatment(s) and are considered ‘treatment resistant’. Although there is no universally accepted definition of treatment-resistant depression (TRD), the most frequently used criterion is the failure to respond to two trials of pharmacological therapy of adequate dose and duration, in the current episode (Fekadu et al., 2018). Less commonly, failure of psychological therapies is also considered to contribute towards the definition of treatment resistance (Conway et al., 2017). The most widely used staging model of TRD is the Thase and Rush model (Thase and Rush, 1997). In this model, failure to respond to one adequate antidepressant trial from a major antidepressant class is considered Stage I TRD (Thase and Rush, 1997), and those who then do not respond to a second adequate antidepressant trial (from a different class than the antidepressant used in Stage I) are termed Stage II TRD (Thase and Rush, 1997). There are variations between measures, studies and groups in terms of requiring a second antidepressant to be from a different class, however. It has been suggested that permitting two ‘failed’ treatments from within a class, and permitting psychological treatments, should be incorporated in updated definitions of Stage II TRD (Rybak et al., 2021).

A recent meta-analysis of augmentation strategies for TRD using the definition of two failed treatments (FTs) identified only 36 randomised controlled trials (RCTs) of pharmacological therapies for qualitative synthesis, of which only 27 were suitable for network meta-analysis (Carter et al., 2020). Similar results were reported in a previous meta-analysis, using the same inclusion criteria (Strawbridge et al., 2019). Only three psychological trials were identified. High pre-post effects were evident across several interventions, albeit in the presence of high between-study heterogeneity. N-methyl-D-aspartate (NMDA)-targeting agents, which are not included as first-line augmenters in treatment guidelines (Taylor et al., 2020), elicited higher effect sizes (ESs) than other classes and with lower heterogeneity (Carter et al., 2020; Strawbridge et al., 2019).

However, this TRD definition does not capture the large proportion of clinical trials of adjunctive treatments in MDD that utilise an inclusion criterion of non-response to one adequate treatment in the current episode. This is clinically significant as non-responders to first-line treatment are at increased risk of non-response to subsequent treatments and poorer long-term functional outcomes (Schosser et al., 2012; Souery et al., 2007). Moreover, in large pragmatic trials, such as STAR*D, approximately one in every two patients with MDD do not respond to initial antidepressant treatment (Rush et al., 2006). Therefore, they represent a large, clinically important population in whom further study of treatment efficacy is necessary. Additionally, to our knowledge, ketamine treatments have not been subjected to meta-analysis in this population of individuals with early-stage TRD. We acknowledge that there are various considerations being made around the terminology used to refer to this population of people. The most common name still in use here is Stage I of the Thase and Rush model of treatment resistance. However, neither this nor other validated staging models incorporate non-response to psychological therapies in their definitions, despite there being arguments in favour of this (Rybak et al., 2021). In order to mirror the substantial previous research using the term ‘TRD’ (Carter et al., 2020; Strawbridge et al., 2019) but to differentiate this article’s definition from TRD defined as non-response to two therapies, we henceforth use the term ‘early-stage TRD’ in reference to a non-response to one adequate pharmacological or psychological therapy for depression. Although we use the term early-stage TRD, which we believe does describe a clinically important and distinct group, we do not suggest that this cohort necessarily follows a linear progression into Stage II TRD (Thase and Rush, 1997).

Given the great number of patients for whom initial monotherapy is not adequately effective, as well as the large number of studies investigating augmentation after one FT (which we term ‘early-stage TRD’ henceforth), it was considered prudent and necessary to evaluate augmentation and combination strategies in patients for whom first-line antidepressant treatment had been ineffective (Stage I TRD, using Thase and Rush’s (1997) staging criteria).

Although the terms augmentation and combination in depression are sometimes used interchangeably today, classically, the combination was used to refer to using two antidepressant medications (or medication and psychological therapy) in tandem, whereas augmentation was the addition of a medication, which was not considered an antidepressant to an antidepressant (e.g. thyroid hormone augmentation) following partial or non-response to an adequate treatment trial (Fava and Targum, 2007). For this systematic review and meta-analysis, however, we have defined augmentation as the addition of any therapy (pharmacological or psychological) to an established continuation treatment, and the combination as the simultaneous commencement of two pharmacological agents or one medication and one psychological therapy. Although this is not a commonly employed strategy (Cleare et al., 2015), it is used clinically (e.g. combined olanzapine and fluoxetine (OFC)) (Luan et al., 2017), and is quite commonly employed as an approach in clinical trials. Our current definition therefore permits a more inclusive investigation of adjunctive treatments for this illness.

The most recent pairwise meta-analysis of the efficacy of pharmacological augmentation strategies in early-stage TRD included studies published up until December 2013 (Zhou et al., 2015). In this 2013 network meta-analysis, TRD was defined as one historical treatment failure, and failure to respond to at least one antidepressant during the current episode. In total, 48 trials (comprising 6654 participants) of 11 augmentation agents were included, and the results for efficacy demonstrated that quetiapine, aripiprazole, thyroid hormone and lithium were all significantly more effective than placebo. In terms of acceptability, which was defined as all-cause discontinuation, there were no significant differences between any of the active agents or with placebo. For tolerability (side-effects discontinuation), quetiapine, olanzapine, aripiprazole and lithium were all significantly less well tolerated than placebo.

By using a more inclusive definition of early-stage TRD (non-response to one adequate course of antidepressant monotherapy), in addition to including psychological and combination treatments, we hope to strengthen the pooled evidence with a substantially greater number of studies and participants than previous meta-analyses of this topic.

The study aims to assess the impact of treatment intervention for patients with early-stage TRD. The objectives were to evaluate treatment improvement in depression, alongside acceptability and tolerability using pre-post treatment effects. Using pre-post effect meta-analyses, we were able to compare effectiveness estimates for heterogeneous treatment strategies and did not require a common comparator (e.g. placebo arm) (Strawbridge et al., 2019). Furthermore, it has been demonstrated that pre-post ESs can estimate the magnitude of treatment effects appropriately, reflecting naturalistic clinical outcomes more closely and also take into account non-specific clinical effects (Bandelow et al., 2015).

## Methods

The protocol for this systematic review was pre-registered (PROSPERO: CRD42018117366) and is described in adherence with the Preferred Reporting Items for Systematic Review and Meta-Analyses (PRISMA) guidelines (Moher et al., 2015). The full search and extraction strategy is described below. Studies were included based on the following a priori eligibility criteria:

### Study designs

We included RCTs that assessed at least one augmentation or combination treatment (with sample sizes of 10 or more). To avoid duplication of data, where multiple manuscripts described one RCT, the eligible comparison with the largest sample size was included.

### Participants

Adults (aged ⩾18 years old) with MDD who had failed to remit despite at least one adequate antidepressant monotherapy trial were included. MDD was defined using either validated rating scales (e.g. the Hamilton Rating Scale for Depression; Hamilton, 1960) or operationalised diagnostic criteria (e.g. the Diagnostic and Statistical Manual of Mental Disorders; American Psychiatric Association, 1994). In keeping with previous meta-analyses, we considered an adequate antidepressant trial to be at least 4 weeks of treatment at recognised minimum-effective doses (where available) (Carter et al., 2020; Strawbridge et al., 2019). Inadequate response to both pharmacological and psychological therapies was permitted, consistent with previous meta-analyses on this subject (Carter et al., 2020; Strawbridge et al., 2019) and in keeping with standardised staging of TRD (Fekadu et al., 2018). RCTs in which the participant population contained ⩾10% of patients with diagnoses of either bipolar or psychotic depression were excluded because of accepted differences in treatment approaches (Cleare et al., 2015).

### Interventions

Studies were eligible for inclusion if participants were randomised to at least one condition where either their current continuation therapy was augmented by addition of a second intervention, or simultaneous commencement of two interventions (two pharmacological agents or one pharmacological and one psychological therapy). For both pharmacological continuation and augmentation agents, treatments included in the Maudsley Treatment Inventory (Fekadu et al., 2018) were permitted, in addition to pharmacological therapies which had reached significance in at least one meta-analysis for depression.

Psychological therapies from the National Institute for Health and Clinical Excellence (NICE) (NICE, 2009) guidelines, or those which had reached meta-analysis significance for treating MDD were deemed eligible (Strawbridge et al., 2019). We made the decision to exclude neurostimulatory treatments, such as electroconvulsive therapy (ECT), transcranial magnetic stimulation and transcranial direct current stimulation, as exploration of these was considered beyond the scope of this review. Pharmacological or psychological comparators were examined in the review (i.e. pill placebo, a different pharmacological treatment, another psychological therapy, waiting list or treatment as usual (TAU)) although other physical treatment comparators (e.g. ECT) were not considered in the current review.

### Outcome measures

Studies were eligible for inclusion if they reported clinical improvement of depression following treatment.

**Primary outcome:** Our primary outcome measure was clinical improvement of depression (or depression symptoms) using validated instruments, summarised with an ES between pre- and post-treatment time points for all eligible treatment and comparator arms. We selected one efficacy instrument per study, giving preference to clinician-rated measures of depression severity. If this was not available, a patient-rated depression severity scale or an assessment of global improvement was reported.

**Secondary outcomes:** We reported any measure of treatment adherence (e.g. participant drop-out due to any cause or specific treatment adherence information) and treatment tolerability (e.g. data on side effects or adverse events) where this data was provided.

### Search strategy

MEDLINE and the Institute for Scientific Information Web of Science electronic databases were searched, in addition to citation lists from included articles. The decision to only use these two databases was made in order to increase the feasibility and optimise the timescale for this study, and the quality of our search was checked and supplemented using handsearching of relevant articles and previously published reviews. For searches using the above-described databases, the following medical subject headings or text word terms were used: (resistan* OR refractor* OR non-respon* OR nonrespon* OR un-respon* OR unrespon* OR TRD OR fail* OR inadequate OR difficult OR intractable[Title/Abstract]) AND (treatment OR intervention OR trial[Title/Abstract]) AND (randomi* OR RCT[Title/Abstract]) AND (combin* OR co-administ* OR augment* OR adjunct* OR add-on[Title/Abstract]) AND (depress* OR MDD OR major depress*[Title/Abstract]). There were no language or date restrictions; searches were conducted for any date up to May 2020.

Search results were independently evaluated against inclusion and exclusion criteria by paired review authors (F.S., S.G., L.M., E.H., C.E, L.K., R.W.T. and J.D.). Any disagreements were resolved in consultation with senior review authors (R.S., A.J.C. and A.H.Y.). Data extraction was performed by a single author for included studies with the extraction data checked independently by a second author (review authors as initialled above). Any discrepancies were resolved by discussion between extracting and reviewing author. Where agreement could not be reached, the senior authors were consulted (as above).

### Risk of bias (RoB) in individual studies

The methodological quality was assessed using the Cochrane RoB tool (Higgins et al., 2022). Using this tool, nine domains were assessed: appropriate and clearly focused research question, allocation sequence randomly generated, allocation adequately concealed, knowledge of allocation adequately prevented, group comparability at baseline ensured, differences among multiple sites adequately addressed (if applicable), selective outcome reporting avoided, intention-to-treat analysis applied and presence of for-profit bias (allegiance). Studies were assessed by two authors, and a RoB rating (high, low or unclear) given for each of the categories above. Disagreements were resolved by senior authors. Each study was then assigned an overall RoB rating of low, moderate or high based on previous criteria (Carter et al., 2020; Strawbridge et al., 2019).

### Measures of treatment effect

Continuous data that described treatment effectiveness were extracted (e.g. pre- and post-severity scores or longitudinal change in severity scores) and presented as a standardised mean difference (Hedges’ g ES). Using a random-effects model, meta-analyses computed a pooled ES with 95% confidence intervals (CIs) and the *I*^2^ statistic. Statistical heterogeneity was considered high if *I*^2^ exceeded 60% (Deeks et al., 2022) and explored using subgroups. The following comparisons were planned to assess the primary outcome:

(a) Pooled effects of augmentation or combination intervention/comparator categories (i.e. psychological treatment, psychological comparator, pharmacological treatment and pharmacological comparator)
(b) Pooled effects of augmenters by class (e.g. selective serotonin reuptake inhibitor (SSRI), serotonin–noradrenaline reuptake inhibitor (SNRI), antipsychotic and mood stabiliser)
(c) Pooled effects of individual treatment interventions within above categories.

### Subgroups

The following subgroups were planned to explore heterogeneity: study quality (RoB), trial duration, stage of treatment resistance (defined by number of FT trials), depression severity, comorbidities, episode duration, continuation treatments and treatment setting.

### Secondary analyses

In terms of secondary outcomes, we explored quantitatively (where possible) or qualitatively: acceptability, tolerability and pair-wise active control comparisons. This final comparison was performed to provide an indicated effect of the treatment and comparator trial arms, which is the current gold standard (Cuijpers et al., 2017).

### Changes since protocol registration

It was originally planned for data from all included studies to be extracted independently by paired authors and then discrepancies assessed. However, considering the large number of included studies, the protocol was amended so that data were extracted by one author, and then independently reviewed for accuracy by a second author. This was decided in order to complete the review in a timely manner, so that the findings accurately reflected the current evidence base. For this reason, pairwise meta-analyses were also not undertaken. Due to the increased heterogeneity in previous analyses of class- and modality-level analyses (see Strawbridge et al., 2019), these were not undertaken. Due to the number of studies and extent of heterogeneity between included study methodologies, the planned subgroups of depression severity, comorbidities, episode duration, continuation treatments and treatment setting were not ultimately considered in analyses.

## Results

### Search results

**Figure 1.**
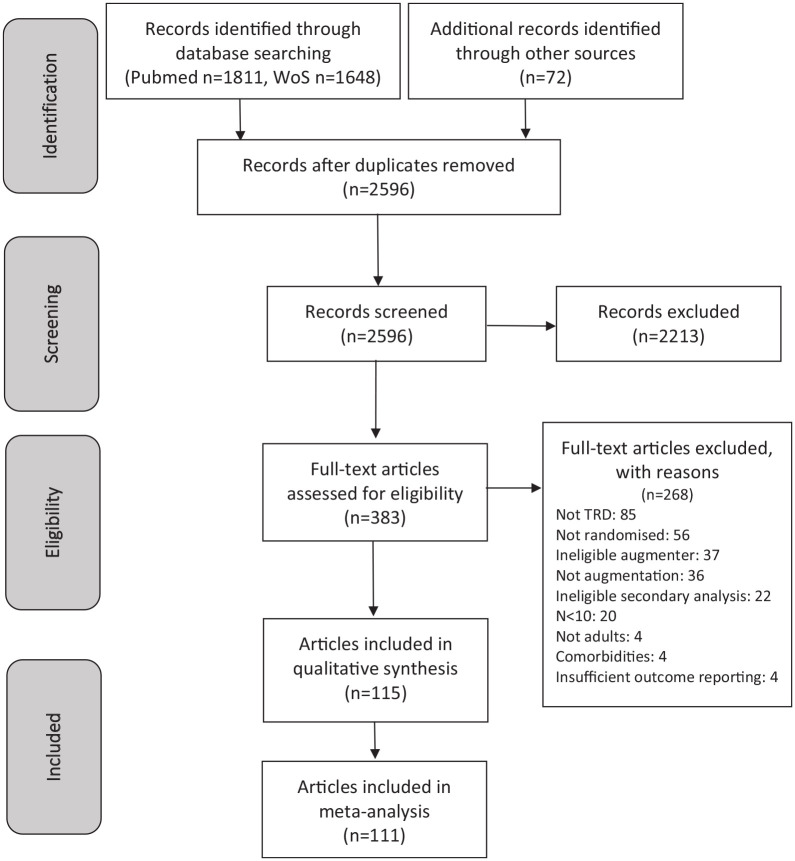
PRISMA flow diagram. TRD: treatment-resistant depression.

The search yielded a total of 3531 records. After removing duplicates, 2587 full texts were reviewed. Of those, 115 articles were included in our narrative review and 111 in our meta-analysis. Figure 1 contains a PRISMA flowchart detailing this search.

### Characteristics of included studies

The characteristics of included studies can be found in Supplemental Table S1. A total of 21,172 participants were included from 115 studies in the narrative synthesis. The mean sample size was 184 participants (median = 80, range = 13–1011). Definitions of TRD varied across studies, frequently using less strict criteria, that is, 68 studies (61%) allowing participants with as few as one FT to be enrolled as TRD. A further 12 (11%) of studies defined TRD as two FTs with either or both lasting less than 6 weeks, whilst only 32 (29%) of included studies met/exceeded the standard criteria for treatment resistance (two FTs in the current episode for a minimum of 6 weeks). Only 7% (*n* = 8) studies specifically stated that the two sequential treatments had to be from different antidepressant classes. Severity, or stage, of treatment resistance is included in Supplemental Table S1.

The median study duration was 6 weeks, with a mean of 9 weeks and a range of 5 days to 18 months. Most included studies took place in North America (53%), with Europe and Asia also well represented (21% and 15%, respectively). Approximately 10% of included studies took place across multiple continents, with North America and Europe the most frequent combination. There was a paucity of data from South America, with only two studies (2%) taking place there (both in Brazil), and no studies taking place in Africa.

### Study participant characteristics

Of the studies reporting participant age (*n* = 111), the mean study age was 46 years (SD = 6), with a range of 28–74. Sex was reported in 106 of the included studies, with the proportion of female participants ranging from 16% to 85% (mean = 63%, SD = 12%).

### Study quality and RoB

Supplemental Table S2a contains the RoB ratings across criteria and studies. RoB ratings were mostly low (*n* = 57) to moderate (*n* = 51), with only 5% (*n* = 6) studies being adjudged to have a high RoB. The RoB criterion most commonly identified as present was ‘allegiance’, likely due primarily to the potential conflict of interest between pharmaceutical companies funding research into their medications. Supplemental Table S2b outlines the mean RoB ratings stratified by treatment class studied.

### Primary outcomes

**Figure 2.**
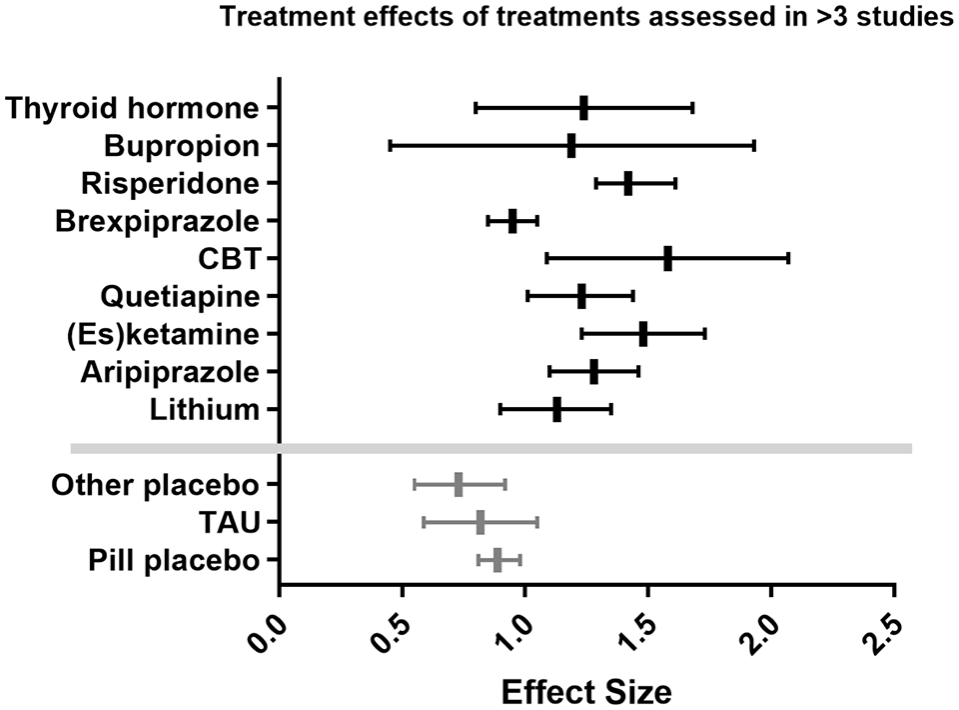
Forest plot displaying ESs for treatments examined in >3 studies.

Table 1 details the results of the meta-analyses, with subgroup analyses investigating TRD severity and duration of treatment presented in Supplemental Tables S3 and S4, respectively. Below the meta-analysis results for the most frequently investigated interventions are described. Figure 2 also presents the results for treatments studied in >3 studies as a forest plot.

**Table 1.** Results of meta-analyses detailing intervention-level data. Standard error, 95% CIs and *I*^2^ heterogeneity are also reported. This table includes studies regardless of TRD severity or treatment duration (which differed in their contribution to heterogeneity) but excludes high RoB and combination studies, which added heterogeneity to analyses.

Modality	Class	Treatment	*k*	*n*	ES	SE	95% CI	*I* ^2^
Antidepressants	SSRI	Citalopram	1	52	2.45	0.28	1.91–3.00	n/a
		Fluoxetine	1	12	0.79	0.33	0.14–1.43	n/a
		Paroxetine	1	5	1.39	0.63	0.16–2.62	n/a
		Sertraline	1	5	0.70	0.5	−0.28 to 1.68	n/a
	TCA	Desipramine	2	26	0.69	0.22	0.26–1.12	0%
	NASSA	Mirtazapine^a^	2	224	1.19	0.09	1.02–1.36	0%
		Mianserin	1	18	1.8	0.38	1.06–2.56	n/a
	Other	Bupropion^b^	4	861	1.19	0.38	0.45–1.93	98%
		Trazodone	1	47	1.67	0.23	1.23–2.12	n/a
Antipsychotics	Typical	Thioridazine	1	38	3.06	0.39	2.30–3.81	n/a
	Atypical	Aripiprazole	12	1971	1.28	0.09	1.10–1.46	86%
		Brexpiprazole	5	1216	0.95	0.05	0.85–1.05	58%
		Cariprazine	2	693	1.11	0.12	0.88–1.34	76%
		Olanzapine	3	220	1.27	0.09	1.09–1.46	0%
		Quetiapine^b^	6	984	1.23	0.11	1.01–1.44	80%
		Risperidone	5	300	1.42	0.17	1.29–1.61	72%
		Ziprasidone	2	112	0.85	0.17	0.52–1.18	54%
Mood stabilisers		Lamotrigine	2	64	1.11	0.16	0.80–1.42	0%
		Lithium^b^	13	430	1.13	0.12	0.90–1.35	50%
		Sodium valproate	1	39	1.63	0.24	1.15–2.11	n/a
Stimulants		Lisdexamfetamine	2	648	0.86	0.05	0.77–0.95	0%
		Methylphenidate	1	72	1.28	0.16	0.97–1.59	n/a
		Modafinil	1	68	1.26	0.16	0.94–1.57	n/a
		Pramipexole^a^	1	30	1.03	0.23	0.59–1.47	n/a
Hormones		Testosterone	3	73	0.73	0.02	0.47–0.99	0%
		Thyroid^b^	4	103	1.24	0.23	0.80–1.68	62%
NMDA		D-cycloserine	2	29	1.40	0.26	0.89–1.92	0%
		Ketamine^c^	8	577	1.48	0.13	1.23–1.73	74%
		Minocycline	1	16	1.59	0.38	0.85–2.33	n/a
Vitamins		L-methylfolate	1	53	1.04	0.17	0.70–1.38	n/a
		SAMe	2	157	1.58	0.12	1.34–1.81	0%
Other/multiple		Buspirone	3	383	1.08	0.22	0.64–1.51	85%
		Buprenorphine^d^	3	192	0.89	0.24	0.42–1.37	79%
		OFC	2	389	1.41	0.33	0.75–2.06	95%
		Mecamylamine	2	501	1.35	0.33	0.70–1.99	47%
		Metyrapone	1	69	0.63	0.13	0.37–0.89	n/a
		Pindolol	3	72	0.81	0.14	0.55–1.08	0%
		Riluzole	1	25	0.45	0.12	0.22–0.68	n/a
Psychological		CBT (inc CT)^e^	6	345	1.58	0.25	1.09–2.07	89%
		DBT	1	10	1.16	0.41	0.36–1.96	n/a
		IPT	1	16	0.93	0.3	0.35–1.52	n/a
		ISTDP	1	39	1.52	0.24	1.06–1.98	n/a
		LTPP	1	53	0.59	0.15	0.30–0.89	n/a
		MBCT	1	67	1.29	0.17	0.96–1.61	n/a
Control		Active psychological^b^	1	86	0.84	0.15	0.56–1.13	n/a
		Other placebo^f^	10	441	0.73	0.10	0.55–0.92	65%
		Pill placebo^b^	57	5606	0.89	0.04	0.81–0.98	82%
		TAU^g^	10	454	0.82	0.12	0.59–1.05	69%

*k*: number of studies; *n*: number of participants; ES: effect size, SAMe: S-adenosyl-L-methionine, CBT: cognitive behavioural therapy, DBT: dialectical behavioural therapy, IPT: interpersonal therapy, ISTDP: intensive short-term dynamic psychotherapy, LTPP: long-term psychodynamic psychotherapy, MBCT: mindfulness-based cognitive therapy, TAU: treatment as usual.

a ES decreased and heterogeneity increased when adding combination study.

b No effect on *I*^2^/ES when adding combination study.

c Four esketamine, one oral, four intravenous (IV), three high-TRD (all IV). ES increased and *I*^2^ decreased slightly when adding combination (ECT) arm. No heterogeneity between the three TRD studies (ES = 1.45) or the oral plus IV studies (ES = 1.5), but much heterogeneity between lower TRD esketamine studies (ES = 1.49, *I*^2^ = 87%).

d When removing Fava et al. (2016), which employed a higher dose and longer duration, heterogeneity reduced to 0% as did ES (0.64; SE = 0.09; 95% CI: 0.46–0.82).

e One high TRD, three digital/blend CBT, one CT. ES and *I*^2^ decreased when adding two combination study. Heterogeneity not affected strongly by format, that is, digital or blended, or CT versus CBT.

f Four nasal, four IV/injection, two gel. *I*^2^ reduced to zero across four IV placebo studies with no effect on ES; *I*^2^ reduced to 42% across two gel placebo studies with a lower ES.

g ES decreased and heterogeneity increased when adding combination.

#### Pharmacological interventions

Antidepressant medications showed a wide range of ESs, with only desipramine (*k* = 2: ES = 0.69, 95% CI: 0.26–1.12, *I*^2^ = 0%), mirtazapine (*k* = 2: ES = 1.19, 95% CI: 1.02–1.36, *I*^2^ = 0%) and bupropion (*k* = 4: ES = 1.19, 95% CI: 0.45–1.93, *I*^2^ = 98%) assessed in more than one study.

All atypical antipsychotics had been assessed in more than one study, with the most common being aripiprazole (*k* = 12: ES *=* 1.28, 95% CI: 1.10–1.46, *I*^2^ = 86%), brexpiprazole (*k* = 5: ES = 0.95, 95% CI: 0.85–1.05, *I*^2^ = 58%) and quetiapine (*k* = 6: ES = 1.23, 95% CI: 1.01–1.44, *I*^2^ = 80%). As with antidepressant medications, the higher *n* studies provide more clustered ESs. Heterogeneity was substantial for nearly all atypical antipsychotics.

Lithium was the most utilised active treatment in the included studies (*k* = 13), forming the vast bulk of mood stabiliser augmentation studies (lamotrigine *k* = 2, sodium valproate *k* = 1). The effect of lithium was found with moderate heterogeneity (ES = 1.13, 95% CI: 0.90–1.35, *I*^2^ = 50%).

Of the less studied treatment modalities, the NMDA modulator ketamine was moderately well investigated (*k* = 8: ES = 1.48, 95% CI: 1.23–1.73, *I*^2^ = 74%). The substantial heterogeneity was explained by the esketamine studies (which recruited participants with less severe treatment resistance); the remaining were one oral and three IV ketamine studies, of which four recruited patients with more severe TRD (*k* = 5: ES *=* 1.50, 95% CI: 1.30–1.71, *I*^2^ = 0%), whereas the four intranasal esketamine studies retained considerable heterogeneity (ES = 1.49, *I*^2^ = 87%), which was not explained, so this finding should be interpreted with caution.

The other pharmacological intervention assessed in >3 studies were thyroid hormones (triiodothyronine and thyroxine) (*k* = 4: ES = 1.24, 95% CI: 0.80–1.68, *I*^2^ = 62%).

#### Psychological interventions

Psychological therapies were examined relatively infrequently as augmentation therapies (*k* = 11). The only therapy to be studied in more than one article was cognitive behavioural therapy, which displayed a high ES in the presence of considerable heterogeneity (*k* = 6: ES = 1.58, 95% CI: 1.09–2.07, *I*^2^ = 89%).

#### Placebo conditions

Effects of pill placebo were comparable to several interventions (*k* = 57, ES = 0.89, 95% CI: 0.81–0.98, *I*^2^ = 82%) although more studies were included. When grouped, other forms of placebo (i.e. nasal spray, IV/injection and gels) were minimally less effective than pill placebo (*k* = 10, ES = 0.73, 95% CI: 0.55–0.92, *I*^2^ = 65%) and TAU performed similarly (*k* = 10, ES = 0.82, 95% CI: 0.59–1.05, *I*^2^ = 69%).

#### Combination interventions

With the exception of OFC, each treatment combination (i.e. two intervention/controls initiated simultaneously) had only been assessed in one study per combination and with wide ranging ESs (presented in Supplemental Table S3). We conclude that, given the methodological differences between these, no conclusions can at present be made about specific combinations from RCTs of people with early-stage TRD.

### Secondary outcomes

#### Subgroup analyses

Additional analyses stratifying between early-stage and substantive TRD criteria required for inclusion in each study are presented in Supplemental Table S4. For all treatments studied, ES 95% CI had large overlap when comparing between studies defining TRD as 1 FT and 2 FTs, suggesting treatment efficacy was not sensitive to TRD definition. The exception to this rule was buspirone, for which the ES and 95% CIs were considerably higher when TRD was defined as 2 FTs (1 FT = ES = 0.84, 95% CI: 0.61–1.07; 2 FTs = ES *=* 1.57, 95% CI: 1.14–2.02), although this consisted of only one small study.

Additional analyses stratifying treatment ESs by the duration of study treatment are presented in Supplemental Table S5. As previously mentioned (Strawbridge et al., 2019), study durations were defined as ‘short-term’ (<6 weeks), ‘adequate duration’ (6–12 weeks) and ‘long-term’ (>12 weeks). Perhaps surprisingly, subgroups of ‘short-term’ and ‘adequate duration’ studies did not result in any consistent findings concerning treatment efficacy. Although approximately half of the treatments studied in this subgroup analysis saw an ES increase when focussing on those studied at an ‘adequate’ duration, half did not. Moreover, 95% CIs and *I*^2^ statistics demonstrated considerable overlap and high heterogeneity, respectively.

#### Tolerability and acceptability

Tolerability data were recorded by 79% of included studies (*k* = 91). The specific measures used were too heterogeneous to allow for meaningful comparison. Data on acceptability were available for 84% of studies (*k* = 97). The most commonly used measure to assess tolerability was dropout due to any cause, which was reported in 28% of active treatment patients, compared to 12% of those receiving placebo.

Dropouts due to adverse events were recorded in 23 articles, returning a dropout rate approximately twice as high in active treatment conditions compared to placebo (9.2% vs 4%). Other, less commonly used, measures included treatment-emergent adverse events, dropout due to intolerance and mean retention time in weeks.

## Discussion

This systematic review and meta-analysis presents an updated current picture of the efficacy of accepted augmentation treatments for primarily early-stage TRD. This synthesis of 115 studies (spanning 41 pharmacological agents and 7 psychological therapies) is a substantial expansion from Zhou et al.’s (2015) meta-analysis of 48 studies (11 augmentation medications). Thus, here we have provided a comprehensive synthesis of the current evidence for TRD. Particular efficacy was apparent for aripiprazole, (es)ketamine, mirtazapine, olanzapine, quetiapine, risperidone and CBT.

### TRD intervention: Still under-researched

The most studied pharmacological augmentation agents were aripiprazole, brexpiprazole, ketamine, lithium and quetiapine, which have each been assessed in at least five studies. However, only the antipsychotics aripiprazole, brexpiprazole and quetiapine have been investigated in at least 1000 patients. OFC was the only combination treatment, which had been consistently investigated. Psychological therapies, with the exception of CBT, do not appear to have been robustly researched as augmentation strategies in early-stage TRD. Likewise, a considerable number of pharmacological therapies have only been investigated in a single trial, often with small numbers of participants, which limits interpretation of real-world clinical efficacy. The authors acknowledge that we were limited by not systematically searching all potentially fruitful databases. The relative paucity of evidence for early-stage TRD is striking, given the marked prevalence of depression, and the high proportion of people not responding to initial antidepressants (Rush et al., 2006; WHO World Mental Health Survey Consortium, 2004). In the largest meta-analysis of treatments for depression to date, Cipriani et al. (2018) included more than 500 studies of 21 antidepressants, with over 115,000 participants. Despite inclusion of only half as many antidepressant agents, Cipriani et al. (2018) were able to identify substantially more suitable clinical trials for depression than we were for early-stage TRD.

More specifically, many potential adjunctive treatments were assessed in only one study (45% of active interventions meta-analysed) and for some, no eligible studies were included at all. An example here is SNRI medications, which are known to be effective as monotherapies (Cleare et al., 2015).

### Statistical efficacy and heterogeneity of clinical outcomes

Of pharmacological augmentation strategies with a reasonable evidence base, which we defined as ⩾2 studies totalling ⩾200 patients, the following (as noted above) had 95% CIs not overlapping with pill placebo’s: aripiprazole, (es)ketamine, mirtazapine, olanzapine, quetiapine, risperidone and CBT. CBT’s was the highest ES of these, at over 1.5. Other reasonably studied agents with high ES, but wider CIs, included mecamylamine and OFC (within-subjects ES > 1.25), bupropion, buspirone, cariprazine and lithium.

However, we report considerable heterogeneity for nearly all identified augmentation approaches, with the exception of brexpiprazole, lithium, mecamylamine, olanzapine and lisdexamfetamine (albeit it with a lower ES). These considerable between-study differences limit the clinical generalisability of the information presented in this review. Expanding on this, we would urge caution in the direct clinical application of the meta-analytical findings presented in this work. The real-world representation of such considerable heterogeneity is likely to be marked differences in response between patients (i.e. a ‘one-size fits all’ approach may not yield the best results).

### Methodological challenges of ketamine

In keeping with a previous meta-analysis of TRD defined as two failed antidepressant trials (Strawbridge et al., 2019), we report the greatest ES for (es)ketamine. Other NMDA receptor modulators (D-cycloserine and minocycline) were also found to perform well, despite wide CIs and smaller sample sizes. However, given its dissociative effects, blinding during studies of ketamine can be difficult to maintain (Acevedo-Diaz et al., 2020) despite ostensibly double-blind trials (Ochs-Ross et al., 2020). Although in our review we find that dissociation is reported with greater frequency in the (es)ketamine group in some studies (Fedgchin et al., 2019; Popova et al., 2019), generally, tolerability is poorly reported across investigations of (es)ketamine (Ionescu et al., 2019; Su et al., 2017). While our findings suggest that further exploration of the NMDA-pathway for potential therapeutic options for TRD is a worthwhile approach, one concern with (es)ketamine is the potential for rapid relapse following treatment cessation, with a mean time to relapse of as short as 6.8 days reported by Diazgranados et al. (2010). However, more recent investigations of ketamine have been more promising, with 30% remaining in remission after 12 months (with a median duration until relapse of 61 days) in one study (Ekstrand et al., 2021). We were not able to examine this fully here.

### Psychological versus pharmacological augmentation

Overall, psychological therapies demonstrated broadly similar ESs to pharmacological augmentation, with CBT in fact demonstrating the greatest ES of any approach. However, with the exception of CBT, we identified a marked scarcity of evidence for psychological therapies in resistant depression. Using a more inclusive definition of early-stage TRD, we were able to present evidence from 12 trials, which is substantially greater than the three studies included in Strawbridge et al.’s (2019) meta-analysis, which used a more stringent definition of TRD (two failed antidepressant trials). What is clear is that further expansion of the evidence for psychological therapies is necessary and is likely to emerge in the coming years (Holmes et al., 2018). It is of note that psychological therapies did not display a higher RoB than other treatment categories; however, as we have previously documented, psychological therapies differ methodologically from medication trials, with components that may inflate treatment efficacy. For example, very rarely participants are blinded to randomisation allocation and allegiance effects reported (Strawbridge et al., 2020).

### Sources of variability assessed

RoB was variable across included investigations, with the vast majority of studies assessed as being at low or moderate risk. Five studies were judged to be at high RoB, and were excluded from primary meta-analyses, as they were found to almost exclusively increase heterogeneity. Evaluation of augmentation agents by duration of treatment had limited effect on ESs or heterogeneity, except in the case of lithium, where subgroup assessment of short-term durations markedly reduced heterogeneity.

As expected, ES were typically lower in studies where all patients had failed to respond to two antidepressant treatments compared to one treatment. Of note, aripiprazole, ketamine, TAU and CBT appeared to be equally/more effective in the higher-TRD subgroup. Buspirone was markedly more effective, although this was in a single study of <50 participants (Fang et al., 2011).

### Potential sources of variability not examined

Although we examined TRD severity as a dichotomous factor for its potential to influence treatment efficacy, we were neither able to look in detail at the different approaches used to assess treatment resistance (e.g. Thase and Rush (1997) vs Massachusetts General Hospital staging models (Fava, 2003)), nor we categorised studies employing a sequential design or prospective open-label standardised treatment to determine TRD. Relatedly, we did not examine the severity or chronicity of patients’ depressive episodes as potential effect modifiers on clinical outcome.

There are several other factors confounders, which could have impacted ESs. These include blinding (and indeed incidences of unblinding), allegiance effects or statistical methods, as well as the extent of the likely efficacy expectation effect, recruitment sources of patients, trial-specific eligibility criteria and generalisability (clinical or demographic) and treatment delivery. It is important to note that these effect modifiers may even differ between treatments (e.g. newer interventions being subject to different methodologies than older interventions). In traditional between-subjects meta-analyses, these factors are largely accounted for, within each study, by the direct group comparisons. Our comparison of within-subjects ES’ between treatments does not account for this study-to-study variability directly.

### The advantages and disadvantages of within-subjects meta-analysis

To address the above-mentioned drawback of our method, the inclusion of control as well as active intervention arms, and indirect comparison between-treatment effects, goes some way to reducing these issues. However, where methodology differs specifically between treatments studied, this cannot be fully accounted for. Of course, in traditional meta-analyses, this is not accounted for either, as only the interventions that are directly compared can be meta-analysed. Moreover, our utilisation of pre-post analysis may suggest larger ESs due to patient expectations or spontaneous remission (Cuijpers et al., 2017). This may be more significant here than in previous meta-analyses on TRD, which have used a more stringent definition of treatment resistance (Strawbridge et al., 2019), as early-stage TRD could reasonably be assumed to have higher rates of natural remission than TRD. Indeed, this appears to be supported by pill placebo having a lower ES in the two FT subgroup. It is noteworthy that pill placebo had a large ES even in conventionally defined-TRD populations, albeit with marked heterogeneity. The marked heterogeneity of pill placebo further demonstrates the limitations of inferring from multiple studies with diverse methodologies. However, it suggests the possibility of improvement for some patients with TRD without the need for augmentation; our within-subjects analysis also includes the assessment of natural recovery. Given the known delay in emergence of complete treatment effects of antidepressants, which can vary between individuals (Uher et al., 2011), there is also the possibility that the continuation treatment is continuing to exert effects, thus attributing observed improvements to the actions of the augmenting agent may be inaccurate. As discussed earlier, there were both pharmacological and psychological augmentation approaches that demonstrated non-overlapping CIs in comparison to placebo, which is suggestive of significance. However, given the limitations of the statistical approach utilised in our review, this is not interpreted as substantive statistical significance. Although Hedges’ g provides similar estimates to Cohen’s d, which is more widely used, there is no rigid approach to interpreting the size of the effect from these numbers alone – particularly in the case of pre-post comparisons (Lakens, 2013). We conclude that despite its limitations, within-subjects meta-analyses have high clinical applicability, in being closer than traditional methods to what is observed in practice when a patient presents requiring treatment for TRD.

### The importance of co-considering benefits and harms

Tolerability and acceptability were reported using a wide variety of measures, which limits our ability to offer direct comparisons between treatments. Tolerability of pharmacological augmentation agents remains a potential concern in the treatment of TRD, especially given the high rates of relapse and the likelihood of requiring continuation or maintenance treatment. Along with the frequency of adverse events, treatments’ safety is clearly guided by their nature, in terms of severity, longevity, treatability and onset/timing: certain adverse effects may become more problematic over a longer duration than the majority of studies identified in this review (e.g. weight gain associated with some atypical antipsychotics). Our inability to fully demonstrate the nature and extent of harms, as well as benefits, of these treatments, is a limitation of the current work. However, it is clearly important to regard benefits and harms in consideration of one another; for example, one included study identified similar efficacy of bupropion and aripiprazole, but aripiprazole was less well-tolerated, and therefore this study alone would suggest augmentation with bupropion is preferred (Mohamed et al., 2017).

### Current evidence-based guidelines

Reflecting, perhaps, the relative lack of consensus evidence around augmentation strategies in early-stage TRD/TRD, there is variation in the recommendations posited in different guidelines. In their systematic review of the topic, Taylor et al. (2020) offered a thorough comparison of 10 national/international guidelines of pharmacological augmentation in TRD. Six guidelines recommend augmentation following one failed antidepressant treatment, whereas four recommend it after two FTs (Taylor et al., 2020). Several guidelines recommend more than one pharmacological class as first line (Taylor et al., 2020). Atypical antipsychotics are regarded as first-line augmentation strategies in seven guidelines including the British Association of Psychopharmacology (Cleare et al., 2015), Clinical Practice Guidelines in the Spanish NHS (Ministry of Health, Social Services and Equality et al., 2014), NICE (NICE, 2009) and World Federation of Societies of Biological Psychiatry (WFSBP) (Bauer et al., 2015). Lithium is considered first line by five guidelines, and addition of bupropion to an SSRI is suggested by the Maudsley Prescribing Guidelines (MPG) (Taylor et al., 2020, 2021). Despite the lack of robust evidence, thyroid hormone(s) remains widely recommended as either a first- or second-line augmentation approach (Malhi et al., 2015; Taylor et al., 2020, 2021). Likewise, the recommendation of buspirone as a first- or second-line augmentation pharmaceutical has been previously reported as not sufficiently evidenced (Suehs et al., 2008; Taylor et al., 2021).

### Progressing towards optimised evidence-based guidelines

Our demonstration of (es)ketamine being well-studied with high efficacy adds to the current literature (Carter et al., 2020; Strawbridge et al., 2019) indicating the potential for being upgraded in guidelines. It is not currently recommended as a first-line augmentation strategy in major national/international guidelines (Taylor et al., 2020). Its highest position is in the MPG where it is considered a second-line choice (Taylor et al., 2021), but some (older) guidelines do not recommend this yet (e.g. Cleare et al., 2015).

Likewise, bupropion appears effective and well-studied in early-stage TRD (albeit with marked heterogeneity), but is only regarded as a first-line pharmacological augmenter by one of the ~10 major national/international guidelines (MPG) (Taylor et al., 2021). Furthermore, its efficacy, safety and tolerability in MDD have been reasonably demonstrated (Patel et al., 2016). Monotherapeutic bupropion is not currently licenced for MDD in some countries (e.g. UK); therefore, we would cautiously recommend that the licencing status of bupropion in the UK be reconsidered.

Finally, despite a general paucity of evidence overall for psychological therapies, the ES of CBT was greater than for other treatments and although CBT is recommended in national guidelines for treating MDD (NICE, 2009), it is rarely specifically recommended as a therapy for TRD (NICE, 2009). Given the results presented in this work, we suggest reconsideration of this position.

In summary, in this large synthesis of augmentation and combination treatments of pharmacological and psychological treatments for TRD, we find both pharmacological and psychological therapies show larger treatment effects than placebo. Our findings firstly support lithium, aripiprazole and quetiapine as current first-line augmenters for TRD; our findings do not show support for brexpiprazole over these agents, although it is a second-line augmenter in some guidelines (Taylor et al., 2020). We urge large-scale investigations of understudied agents showing promise, including modafinil, S-adenosyl-L-methionine and cognitive behavioural analysis system of psychotherapy. Finally, we hope that our findings are considered in updating treatment guidelines, particularly in the potential for upgrading (es)ketamine, CBT, mecamylamine and bupropion. We acknowledge, however, that our findings are not free from methodological problems and considerable heterogeneity remains between studies for most treatment approaches, in addition to an enduring overall relative paucity of evidence for monotherapy-resistant depression.

## Supplemental Material

sj-docx-1-jop-10.1177_02698811221104058 – Supplemental material for Systematic review and meta-analysis of augmentation and combination treatments for early-stage treatment-resistant depression Click here for additional data file. Supplemental material, sj-docx-1-jop-10.1177_02698811221104058 for Systematic review and meta-analysis of augmentation and combination treatments for early-stage treatment-resistant depression by Fraser Scott, Elliot Hampsey, Sam Gnanapragasam, Ben Carter, Lindsey Marwood, Rachael W Taylor, Cansu Emre, Lora Korotkova, Jonatan Martín-Dombrowski, Anthony J Cleare, Allan H Young and Rebecca Strawbridge in Journal of Psychopharmacology
