# Supplementary material for: Systematic review and meta-analysis of augmentation and combination treatments for early-stage treatment-resistant depression
Source: J Psychopharmacol. 2022 Jul 21;37(3):268–78. doi: 10.1177/02698811221104058 (PMC10076341; doi:10.1177/02698811221104058)
Supplement: sj-docx-1-jop-10.1177_02698811221104058 – Supplemental material for Systematic review and meta-analysis of augmentation and combination treatments for early-stage treatment-resistant depression [file sj-docx-1-jop-10.1177_02698811221104058.docx]

**Supplementary material**

**Supplementary Table 1: included study characteristics**

| **Class/study reference** | **TRD code** | **N** | **Treatment** | **Dose** | **Duration (weeks)** | **Outcome measure** | **Avg age** | **% female** | **N analysed** | **% responded** | **Tolerability value** | **Tolerability measure (unit)** | **Acceptability Value** | **Acceptability measure (unit)** |
| --- | --- | --- | --- | --- | --- | --- | --- | --- | --- | --- | --- | --- | --- | --- |
| **Antipsychotics** |  |  |  |  |  |  |  |  |  |  |  |  |  |  |
| Astellas Pharma. | 0 | 128 | Quetiapine | 50/150/300mg | 6 | MADRS | 39/35/37 | 34/42/40 | 128 | nr | 10% | Any AE | 7/15/9% | Dropout AE |
|  |  | 44 | Placebo | n/a |  |  | 40 | 43 | 44 | nr | 2% | Any AE | 2% | Dropout AE |
| Bauer et al., 2009 | 1 | 167/ 163 | Quetiapine | 150mg/ 300mg | 6 | MADRS | 46/ 45.5 | 69.3/ 68.3 | 166/ 161 | 55.4/ 57.8 | 0.8/ 1.2 | TEAEs /ppt | 6.6%/ 11.7% | Dropout AE |
|  |  | 163 | Placebo | n/a |  |  | 44.8 | 65 | 160 | 46.3 | 0.4 | TEAEs /ppt | 3.70% | Dropout AE |
| Bauer et al., 2018 | 0 | 444 | Brexpiprazole | 1-3mg titr. | 24 | MADRS | 47.1 | 69.1 | 444 | 21.4 | 0.6 | TEAEs /ppt | 6.30% | Dropout AE |
|  |  | 442 | Placebo | n/a |  |  | 46.4 | 68.5 | 441 | 24.9 | 0.5 | TEAEs /ppt | 3.40% | Dropout AE |
| Berman et al., 2007 | 2 | 182 | Aripiprazole | 5-15mg titr. | 6 | MADRS | 46.5 | 61.5 | 182 | 33.7 | 1 | AEs /ppt | 12.10% | Dropout Any |
|  |  | 176 | Placebo | n/a |  |  | 44.2 | 64.2 | 176 | 23.8 | 0.6 | AEs /ppt | 9.10% | Dropout Any |
| Berman et al., 2009 | 2 | 177 | Aripiprazole | 2-20mg titr. | 6 | MADRS | 45.1 | 78 | 176 | 46.6 | 1 | TEAEs /ppt | nr | nr |
|  |  | 172 | Placebo | n/a |  |  | 45.6 | 68 | 172 | 26.6 | 0.5 | TEAEs /ppt | nr | nr |
| Dunner et al., 2007 | 2 | 22/ 19 | Ziprasidone | 40-80mg / 80-160mg titr. | 8 | MADRS | 43.1/ 42.6 | 54.5/ 47.4 | 21/ 19 | 19.0/ 31.6 | 100/ 84.2% | Any AE | 40.9/ 36.8% | Dropout AE |
| Durgam et al., 2016 | 0 | 274/276 | Cariprazine | 1-2mg / 2-4.5mg titr. | 8 | MADRS | 45.5/ 45.1 | 68.5/ 73.6 | 273/ 271 | 48.0/49.4 | 0.5/ 0.9 | TEAEs /ppt | 13.2/ 3.0% | Dropout AE |
|  |  | 269 | Placebo | n/a |  |  | 46.4 | 71.4 | 264 | 38.3 | 0.1 | TEAEs /ppt | 0.00% | Dropout AE |
| Earley et al., 2018 | 2 | 269 | Cariprazine | 1.5-4mg titr. | 8 | MADRS | 44.2 | 64.7 | 267 | 28.1 | 67.30% | Any TEAE | 8.60% | Dropout AE |
|  |  | 258 | Placebo | n/a |  |  | 43.8 | 65.9 | 258 | 27.5 | 50.80% | Any TEAE | 1.20% | Dropout AE |
| El-Khalili et al., 2010 | 0 | 148/ 150 | Quetiapine | 150/300mg | 8 | MADRS | 45.9/ 44.3 | 76.2/ 72.6 | 143/ 146 | 51.7/ 58.9 | 1.1/ 1.3 | TEAEs /ppt | 11.5/ 19.5% | Dropout AE |
|  |  | 148 | Placebo | n/a |  |  | 46.2 | 68.5 | 143 | 46.2 | 0.6 | TEAEs /ppt | 0.70% | Dropout AE |
| Fava et al., 2012 | 3 | 56 | Aripiprazole | 2mg + various | 8 | MADRS | 45.4 | 66 | 56 | 18.5 | 50.40% | Any TEAE | 0.00% | Dropout AE |
|  |  | 169 | Placebo | n/a + various |  |  | 45.1 | 64 | 169 | 17.4 | 47.60% | Any TEAE | 0.00% | Dropout AE |
| Fava et al., 2018 | 2 | 76/74 | Cariprazine | 0.1-0.3/ 1-2 mg | 8 | MADRS | 46.6/ 44.2 | 68.4/ 69.9 | 76/ 73 | 30.3/ 38.4 | 0.29/ 0.2 | TEAEs /ppt | 1.3/ 2.7% | Dropout AE |
|  |  | 81 | Placebo | n/a + various |  |  | 45.2 | 75.3 | 72 | 25.9 | 0.1 | TEAEs /ppt | 25.00% | Dropout AE |
| Han et al., 2015 | 0 | 52 | Aripiprazole | 2-15mg titr. | 6 | MADRS | 47.9 | 70 | 50 | 60 | 71.10% | Any AE | 6.00% | Dropout AE |
| Hobart et al., 2018a | 2 | 197 | Brexpiprazole | 2-3mg titr. + various | 6 | MADRS | 43.6 | 65 | 191 | 10.5 | 50.80% | Any TEAE | 1.00% | Dropout TEAE |
|  |  | 100 | Quetiapine | 150-300mg titr. |  |  | 44.6 | 66 | 99 | nr | 58.00% | Any TEAE | 4.00% | Dropout TEAE |
|  |  | 206 | Placebo | n/a + various |  |  | 41.8 | 72.3 | 205 | 6.8 | 51.90% | Any TEAE | 0.50% | Dropout TEAE |
| Hobart et al., 2018b | 1 | 192 | Brexpiprazole | 2mg | 6 | MADRS | 43 | 76.6 | 192 | 37.5 | 2.10% | TEAE discont | 7.80% | Dropout Any |
|  |  | 202 | Placebo | n/a |  |  | 42.7 | 71.3 | 202 | 32.7 | 0.50% | TEAE discont | 3.00% | Dropout Any |
| Horikoshi et al., 2019 | 0 | 17/ 14 | Aripiprazole | 3mg/ 3-12mg titr. | 6 | MADRS | 38.8/ 44.2 | 47.1/ 57.1 | 17/ 14 | 17.6/ 57.1 | nr | nr | 5.9/ 35.7% | Dropout AE |
| Ionescu et al., 2010 | 0 | 42 | Risperidone | 0.25-2mg titr. | 6 | HAMD-17 | 45 | 73.8 | 42 | nr | 47.60% | Any TEAE | nr | nr |
|  |  | 42 | Placebo | n/a |  |  | 46 | 76.2 | 42 | nr | 52.40% | Any TEAE | nr | nr |
| Kamijima et al., 2013 | 2 | 197/ 194 | Aripiprazole | 3mg/ 3-15mg titr. | 6 | MADRS | 39.2/ 38.1 | 37.1/ 47.9 | 197/ 194 | 42.1/ 39.2 | 0.3/ 0.8 | TEAEs /ppt | 2.5/ 2.6% | Dropout AE |
|  |  | 195 | Placebo | n/a |  |  | 38.7 | 41 | 195 | 28.2 | 0 | TEAEs /ppt | 1.00% | Dropout AE |
| Kamijima et al., 2018 | 0 | 209 | Aripiprazole | 3-12mg titr. | 6 | MADRS | 38.3 | 38 | 208 | 37.5 | 61.20% | Any TEAE | 1.90% | Dropout AE |
|  |  | 203 | Placebo | n/a |  |  | 39.5 | 35.5 | 203 | 25.6 | 53.20% | Any TEAE | 1.50% | Dropout AE |
| Keitner et al., 2009 | 0 | 64 | Risperidone | 0.5-3mg titr. | 4 | MADRS | 45.5 | 57.8 | 62 | 54.8 | 0.52 | AEs /ppt | 12.90% | Dropout AE |
|  |  | 33 | Placebo | n/a |  |  | 44.6 | 54.5 | 30 | 33.2 | 0.45 | AEs /ppt | 21.00% | Dropout AE |
| Lenze et al., 2015 | 0 | 91 | Aripiprazole | 2-15mg titr. | 12 | MADRS | 66.4 | 57 | 91 | 44 | 2.8 | AEs /ppt | 5.50% | Dropout Any |
|  |  | 90 | Placebo | n/a |  |  | 65.7 | 57 | 90 | 28.9 | 2.3 | AEs /ppt | 8.90% | Dropout Any |
| Marcus et al., 2008 | 2 | 191 | Aripiprazole | 2-20mg titr. | 6 | MADRS | 44.6 | 66 | 185 | 32.4 | 0.9 | AEs /ppt | 31.40% | Dropout Any |
|  |  | 190 | Placebo | n/a |  |  | 44.4 | 67.4 | 184 | 17.4 | 0.3 | AEs /ppt | 30.40% | Dropout Any |
| McIntyre et al., 2007 | 0 | 29 | Quetiapine | 50-400mg titr. | 8 | HAMD-17 | 44 | 65 | 29 | 48 | 3.3 | AEs /ppt | 27.60% | Dropout AE |
|  |  | 29 | Placebo | n/a |  |  | 45 | 59 | 29 | 28 | 2.8 | AEs /ppt | 6.90% | Dropout AE |
| Reeves et al., 2008 | 0 | 12 | Risperidone | 0.25-2mg titr. | 8 | MADRS | 46.5 | 92 | 12 | nr | 1.9 | AEs /ppt | nr | nr |
|  |  | 11 | Placebo | n/a + various |  |  | 41.3 | 45 | 11 | nr | 2.6 | AEs /ppt | nr | nr |
| Thase et al., 2015a | 2 | 188 | Brexpiprazole | 2mg | 6 | HAMD-17 | 44.1 | 69.1 | 175 | 15.7 | 59.00% | Any AE | 0.00% | Dropout TEAE |
|  |  | 191 | Placebo | n/a |  |  | 45.2 | 71.7 | 178 | 23.4 | 46.60% | Any AE | 3.20% | Dropout TEAE |
| Thase et al., 2015b | 2 | 226/ 230 | Brexpiprazole | 0.5-1mg./ 0.5-3mg titr. | 6 | MADRS | 45.7/ 44.5 | 69.9/ 67.8 | 211/ 213 | 23.2/ 23.0 | 46.8/ 54.9% | Any AE | 1.3/ 3.5% | Dropout TEAE |
|  |  | 221 | Placebo | n/a |  |  | 46.6 | 66.1 | 203 | 14.3 | 63.30% | Any AE | 1.40% | Dropout TEAE |
| Yoshimura et al., 2012 | 1 | 13/11 | Aripiprazole | 8.73/ 9.23mg (means) | 4 | HAMD-17 | 39.3/ 43.3 | 54.5/ 53.8 | 13/11 | 27.7/ 15.4 | 1.9/ 2.2 | SAS score | nr | nr |

| **Class/study reference** | **TRD code** | | | **N** | | **Treatment** | **Dose** | **Duration (weeks)** | **Outcome measure** | **Avg age** | **% female** | **N analysed** | **% responded** | **Tolerability value** | **Tolerability measure (unit)** | **Acceptability Value** | **Acceptability measure (unit)** |
| --- | --- | --- | --- | --- | --- | --- | --- | --- | --- | --- | --- | --- | --- | --- | --- | --- | --- |
| **Multiple treatment classes** | | | | | | |  |  |  |  |  |  |  |  |  |  |  |
| Adli et al., 2017 | | 0 | 34 | | Lithium | | Serum 0.6-0.8mmol/L | 20 | HAMD-21 | nr | nr | 34 | nr | nr | nr | 29.40% | Dropout Any |
|  |  |  | 1 | | T3 | | 37.5ug |  |  | nr | nr | 1 | nr | nr | nr | 100.00% | Dropout Any |
| Bauer et al., 2013 | | 0 | 231 | | Quetiapine | | 300mg titr. | 6 | MADRS | nr | nr | 231 | 52.4 | 29.90% | TEAE discont | 15.20% | Dropout Any |
|  |  |  | 229 | | Lithium | | 0.6–1.2 mmol/L titr. |  |  | nr | nr | 229 | 46.2 | 7.90% | TEAE discont | 18.30% | Dropout Any |
| Cheon et al., 2017 | | 0 | 56 | | Aripiprazole | | 2.5-20mg titr. | 6 | MADRS-17 | 43.9 | 58.9 | 56 | 60.6 | nr | nr | 25.00% | Dropout Any |
|  |  |  | 47 | | Bupropion | | 150-300mg titr. |  |  | 47.7 | 72.3 | 47 | 42.6 | nr | nr | 23.40% | Dropout Any |
| Doree et al., 2007 | | 0 | 10 | | Quetiapine | | 400mg | 8 | HAMD-17 | 52.3 | 50 | 10 | 80 | 0.00% | TEAE discont | 0.00% | Dropout Any |
|  |  |  | 10 | | Lithium | | Serum 0.78mmol/L titr. |  |  | 49.3 | 70 | 10 | 50 | 20.00% | TEAE discont | 30.00% | Dropout Any |
| Fang et al., 2011 | | 2 | 48 | | T3/T4 | | 80mg | 8 | HAMD-17 | nr | nr | 48 | 58.3 | nr | nr | nr | nr |
|  |  |  | 47 | | Trazodone | | 100mg |  |  | nr | nr | 47 | 61.7 | nr | nr | nr | nr |
|  |  |  | 46 | | Buspirone | | 30mg |  |  | nr | nr | 46 | 56.5 | nr | nr | nr | nr |
|  |  |  | 45 | | Risperidone | | 2mg |  |  | nr | nr | 45 | 46.7 | nr | nr | nr | nr |
|  |  |  | 39 | | Sodium Valproate | | 600mg |  |  | nr | nr | 39 | 61.5 | nr | nr | nr | nr |
|  |  |  | 45 | | Placebo | | n/a |  |  | nr | nr | 45 | 66.7 | nr | nr | nr | nr |
| Fava et al., 1994 | | 0 | 7 | | Desipramine | | 25-50mg titr. | 4 | HAMD-17 | 39.6 | 61 | 7 | 14.3 | nr | nr | 42.90% | Dropout Any |
|  |  |  | 6 | | Lithium | | 300-600mg titr. |  |  | 39.6 | 61 | 6 | 50 | nr | nr | 33.30% | Dropout Any |
| Fava et al., 2002 | | 0 | 19 | | Desipramine | | 25-50mg titr. | 4 | HAMD-17 | 39.9 | 44.1 | 19 | 26.3 | 4.1 | TEAEs /ppt | 14.70% | Dropout Any |
|  |  |  | 16 | | Lithium | | 300-600mg titr. |  |  | 43.9 | 50 | 16 | 12.5 | 3.9 | TEAEs /ppt | 14.70% | Dropout Any |
| Joffe et al., 1993 | | 0 | 18 | | Lithium | | 900-1200mg titr. | 2 | HAMD-17 | 37.4 | 47.1 | 17 | 52.9 | nr | nr | 5.90% | Dropout Any |
|  |  |  | 17 | | Liothyronine | | 37.5ug |  |  | 37.4 | 70.6 | 17 | 58.8 | nr | nr | 0.00% | Dropout Any |
|  |  |  | 16 | | Placebo | | n/a |  |  | 37.4 | 62.5 | 16 | 18.8 | nr | nr | 0.00% | Dropout Any |
| Joffe et al., 2006 | | 0 | 10 | | T3 | | 37.5ug | 2 | HAMD-17 | 42.2 | 11 | 10 | nr | nr | nr | nr | nr |
|  |  |  | 9 | | Lithium | | 600mg |  |  | 38.3 | 89 | 9 | nr | nr | nr | nr | nr |
|  |  |  | 9 | | Lithium + T3 | | 600-900mg titr. + 37.5ug |  |  | 37 | 78 | 9 | nr | nr | nr | nr | nr |
|  |  |  | 8 | | Placebo | | n/a |  |  | 38.8 | 75 | 8 | nr | nr | nr | nr | nr |
| Maes et al., 1996 | | 0 | 13 | | Fluoxetine | | 20mg | 4 | HAMD-17 | 49.6 | 33.3 | 12 | 75 | 0.00% | AE discont | nr | nr |
|  |  |  | 12 | | Pindolol | | 7.5mg |  |  | 61.5 | 45.5 | 11 | 72.7 | 0.00% | AE discont | nr | nr |
|  |  |  | 12 | | Placebo | | n/a |  |  | 56.6 | 70 | 10 | 20 | 0.00% | AE discont | nr | nr |
| Mahmoud et al., 2007 | | 0 | 141 | | Risperidone | | 1-2mg titr. | 6 | HAMD-17 | 45.9 | 70.8 | 137 | 46.2 | 0.5 | TEAEs /ppt | 5.80% | Dropout AE |
|  |  |  | 133 | | Placebo | | n/a |  |  | 46.4 | 76.3 | 131 | 29.5 | 0.5 | TEAEs /ppt | 2.30% | Dropout AE |
| Mohamed et al., 2017 | | 0 | 506 | | Bupropion | | 150-400mg titr. | 12 | QIDS-C16 | 54.4 | 16 | 506 | 65.6 | 2.9 | AEs /ppt | 25.30% | Dropout Any |
|  |  |  | 505 | | Aripiprazole | | 2-15mg titr. |  |  | 54.2 | 15.2 | 505 | 74.3 | 2.8 | AEs /ppt | 19.60% | Dropout Any |
| Navarro et al., 2019 | | 0 | 52 | | Lithium | | 200-600mg titr. | 10 | HAMD-21 | 56.1 | nr | 52 | 21.1 | 0 | SAEs /ppt | 0.00% | Dropout Any |
|  |  |  | 52 | | Citalopram | | 15-30mg titr. |  |  | 54.7 | nr | 52 | 40.4 | 0 | SAEs /ppt | 0.00% | Dropout Any |
| Nierenberg et al., 2006 | | 2 | 73 | | T3 | | 25-50ug titr. | ≤14 | QIDS-SR | 43.2 | 41 | 73 | 24.7 | 80.00% | Any AE | 9.60% | Dropout intolerance |
|  |  |  | 69 | | Lithium | | 450-900mg titr. |  |  | 40.6 | 42 | 69 | 15.9 | 79.40% | Any AE | 23.20% | Dropout intolerance |
|  |  |  | 17 | | Placebo | | n/a |  |  | 39.7 | 41.2 | 15 | 20 | nr | nr | nr | nr |
| Papakostas et al., 2015 | | 0 | 71 | | Ziprasidone | | 40-160mg titr. | 8 | HAMD-17 | 44.7 | 69 | 71 | 35.2 | 1.5 | TEAEs /ppt | 14.10% | Dropout intolerance |
|  |  |  | 68 | | Placebo | | n/a |  |  | 44.2 | 72 | 68 | 20.6 | 1.1 | TEAEs /ppt | 0.00% | Dropout AE |
| Trivedi et al., 2006 | | 0 | 286 | | Buspirone | | 15-60mg titr. | 12 | QIDS-SR | 41.5 | 55.9 | 286 | 30.1 | 77.40% | Any AE | 20.60% | Dropout TEAE |
|  |  |  | 279 | | Bupropion | | 200-400mg |  |  | 40.8 | 61.6 | 279 | 29.7 | 79.70% | Any AE | 12.50% | Dropout TEAE |
| Yoshimura et al., 2014 | | 0 | 10 | | Lithium | | 458mg (mean) | 4 | HAMD-17 | 39 | 60 | 10 | 40 | 20.00% | Any AE | 10.00% | Dropout Any |
|  |  |  | 10 | | Aripiprazole | | 9mg (mean) |  |  | 40 | 70 | 10 | 40 | 20.00% | Any AE | 10.00% | Dropout Any |
|  |  |  | 10 | | Olanzapine | | 7mg (mean) |  |  | 42 | 50 | 10 | 30 | 10.00% | Any AE | 0.00% | Dropout Any |

| **Class/study reference** | **TRD code** | **N** | **Treatment** | **Dose** | **Duration (weeks)** | **Outcome measure** | **Avg age** | **% female** | **N analysed** | **% responded** | **Tolerability value** | **Tolerability measure (unit)** | **Acceptability Value** | **Acceptability measure (unit)** |
| --- | --- | --- | --- | --- | --- | --- | --- | --- | --- | --- | --- | --- | --- | --- |
| **Psychological therapies** |  |  |  |  |  |  |  |  |  |  |  |  |  |  |
| Eisendrath et al., 2016 | 3 | 87 | Mindfulness-based CBT | ~22 hours | 8 | HAMD-17 | 47.1 | 75.9 | 87 | 30.7 | nr | nr | 12.70% | Dropout Any |
|  |  | 86 | HEP | ~22 hours |  |  | 45.2 | 76.7 | 86 | 15.3 | nr | nr | 16.30% | Dropout Any |
| Fonagy et al., 2015 | 1 | 67 | LTPP | ~ 78 hours | 78 | HAMD-17 | 42.7 | 66.7 | 53 | nr | nr | nr | 32.10% | Dropout Any |
|  |  | 62 | TAU | Variable | Variable |  | 46.1 | 66.1 | 46 | nr | nr | nr | 17.40% | Dropout Any |
| Harley et al., 2008 | 0 | 13 | DBT | ~24 hours | 16 | HAMD-17 | 41.8 | 75 | 10 | nr | nr | nr | 30.00% | Dropout Any |
|  |  | 11 | TAU | n/a |  |  | 41.8 | 75 | 9 | nr | nr | nr | 22.20% | Dropout Any |
| Hauksson et al., 2017 | 3 | 86 | CBT (group) | ~ 18 hours | 6 | BDI-II | 43.4 | 83.7 | 83 | nr | nr | nr | nr | nr |
|  |  | 59 | CBT (individual) | ~ 10 hours |  |  | 45.4 | 66.1 | 59 | nr | nr | nr | nr | nr |
|  |  | 36 | TAU | n/a |  |  | 46.1 | 75 | 36 | nr | nr | nr | nr | nr |
| Kocsis et al., 2012 | 0 | 200 | CBASP | 12.5 sessions (mean) | 12 | HAMD-24 | 45.3 | 56 | 174 | nr | 26.20% | Any AE | 1.10% | Dropout AE |
|  |  | 195 | BSP | 13.1 sessions (mean) |  |  | 46.4 | 57.9 | 168 | nr | 27.00% | Any AE | 0.60% | Dropout AE |
| Nakagawa et al., 2017 | 0 | 40 | CBT | ~13.5 hours | 16 | GRID HAMD-17 | 39.5 | 37.5 | 40 | 77.5 | 0.00% | Any SAE | 8.80% | Dropout Any |
|  |  | 40 | TAU | n/a |  |  | 41.7 | 35 | 40 | 32.5 | 0.00% | Any SAE | nr | n/a |
| Nakao et al., 2018 | 0 | 20 | CBT (web & in person) | ~9 hours | 12 | GRID HAMD-17 | 39.7 | 50 | 20 | 55 | 0.00% | Any SAE | nr | nr |
|  |  | 20 | delay CBT + TAU | ~9 hours | 24† |  | 40.6 | 50 | 20 | 20 | 0.00% | Any SAE | nr | nr |
| Souza et al., 2016 | 0 | 17 | IPT | 12 (IPT) + 4.5 (TAU) | 16 to 19 | HAMD-17 | 49.3 | 88.2 | 16 | 35.5 | nr | nr | 37.50% | Dropout Any |
|  |  | 23 | TAU | 4.27 (mean) sessions |  |  | 49.2 | 82.6 | 18 | 22.2 | nr | nr | 11.10% | Dropout Any |
| Thase et al., 2007b | 1 | 65 | CBT | 11.4 (±4.9) sessions | 12 | QIDS-SR | 40.6 | 63.1 | 65 | 35.4 | 66.60% | Any AE | 9.20% | Dropout intolerance |
| Town et al., 2017 | 0 | 30 | STDP | 16.1 (±6.6) sessions | 20 | HAMD-17 | 38.9 | 56.7 | 30 | 36 | 0 | AEs /ppt | 12.80% | Dropout Any |
|  |  | 30 | TAU | n/a |  |  | 44.2 | 70 | 30 | 3.7 | 0.1 | AEs /ppt | n/a | n/a |
| Wiles et al., 2008 | 0 | 14 | CBT | 12-20 sessions (median 9.5) | 12 to 18 sessions | BDI-II | 45.5 | 85.7 | 14 | 57.1 | nr | nr | 0.00% | Dropout Any |
|  |  | 11 | TAU | n/a |  |  | 45.1 | 81.8 | 11 | 0 | nr | nr | 18.20% | Dropout Any |
| Wiles et al., 2013 / 2014 | 0 | 234 | CBT | 12-18 hours (avg 11) | 6.3 months (mean) | BDI-II | 49.2 | 68.8 | 206 | 46.1 | nr | nr | 10.70% | Dropout Any |
|  |  | 235 | TAU | n/a |  |  | 50 | 75.7 | 213 | 21.6 | nr | nr | 9.40% | Dropout Any |

| **Class/study reference** | **TRD code** | | | **N** | | **Treatment** | **Dose** | **Duration (weeks)** | **Outcome measure** | **Avg age** | **% female** | **N analysed** | **% responded** | **Tolerability value** | **Tolerability measure (unit)** | **Acceptability Value** | **Acceptability measure (unit)** |
| --- | --- | --- | --- | --- | --- | --- | --- | --- | --- | --- | --- | --- | --- | --- | --- | --- | --- |
| **Combination treatments** | | | | | | |  |  |  |  |  |  |  |  |  |  |  |
| Chaput et al., 2008 | | 2 | 11 | | Quetiapine + CBT | | 25mg | 12 | MADRS | 41.6 | 73 | 11 | 20 | 2.2 | BAS total /ppt | 0.00% | Dropout AE |
|  |  |  | 11 | | Placebo + CBT | | n/a + ~12 hours |  |  | 44.9 | 73 | 11 | 6 | 0.1 | BAS total /ppt | 0.00% | Dropout AE |
| Corya et al., 2006 | | 2 | 59/ 243 | | OFC | | 1mg + 5mg/ 6-12mg + 25-50mg | 12 | MADRS | 45.7/ 45.7 | 72.5/ 72.5 | 59/ 243 | 36.4/ 43.3 | 3.4%/ 11.9% | AE discont | 20.3%/ 24.7% | Dropout Any |
| Fornaro et al., 2014 | | 0 | 25 | | Duloxetine + Buproprion | | 86.1mg + 215.2mg (means) | 6 | HAMD-21 | 42.6 | 70 | 23 | 26.1 | 21.70% | TEAE discont | 61.00% | Dropout Any |
|  |  |  | 23 | | Duloxetine + Placebo | | 91.3mg (mean) + n/a |  |  | 35.5 | 61 | 22 | 21.7 | 13.00% | TEAE discont | 68.00% | Dropout Any |
| Franco-Chaves et al., 2013 | | 0 | 13 | | Pramipexole + Escitalopram | | 0.375-2.25mg titr. + 10mg | 6 | MADRS | 46.1 | 77 | 13 | 7.7 | 46.20% | TEAE discont | 69.20% | Dropout Any |
| Gamble et al., 2018 | | 0 | 14 | | Ketamine + ECT | | 0.75mg/kg | 2 to 4 | MADRS | 42 | 50 | 12 | 100 | 7.5 | TEAEs /ppt | nr | nr |
|  |  |  | 13 | | Propofol + ECT | | 1mg/kg |  |  | 46 | 50 | 12 | 83.3 | 7.7 | TEAEs /ppt | nr | nr |
| Mantani et al., 2017 | | 0 | 81 | | iCBT + medication switch | | ~2.75 hours + various | 9 | PHQ-9 | 40.2 | 56.8 | 81 | 42.3 | 0 | SAEs /ppt | 19.80% | Dropout Any |
|  |  |  | 83 | | Medication switch | | various |  |  | 41.6 | 49.4 | 83 | 21.2 | 0 | SAEs /ppt | 16.90% | Dropout Any |
| McGrath et al., 2006 | | 3 | 51 | | Venlafaxine + Mirtazapine | | 210.3mg + 35.7mg (means) | 12 | QIDS-SR | 45.3 | 45.1 | 51 | 23.5 | 55.10% | Any AE | 21.60% | Dropout AE |
| Shelton et al., 2001 | | 1 | 10 | | OFC | | 5-20mg + 20-60mg titr. | 8 | MADRS | 42 | 75 | 10 | 60 | 60.00% | AE discont | 10.00% | Dropout Any |
|  |  |  | 10 | | Fluoxetine + Placebo | | 20-60mg + n/a titr. |  |  | 42 | 75 | 10 | 10 | 10.00% | AE discont | 30.00% | Dropout Any |
|  |  |  | 8 | | Olanzapine + Placebo | | 5-20mg + n/a titr. |  |  | 42 | 75 | 7 | 0 | 0.00% | AE discont | 25.00% | Dropout Any |
| Shelton et al., 2005 | | 1 | 146 | | OFC | | 6-12mg + 25-50mg | 7 | MADRS | 42.5 | 67.1 | 146 | 27.5 | 14.60% | AE discont | 20.50% | Dropout Any |
| Stabl et al., 1995 | | 1 | 38 | | Moclobemide + Thioridazine | | 450mg + 100mg | 4 | HAMD-21 | 51 | 90 | 38 | 74 | 21.00% | Any AE | 2.60% | Dropout AE |
|  |  |  | 40 | | Moclobemide + Placebo | | 450mg + n/a |  |  | 53 | 67 | 40 | 77 | 15.00% | Any AE | 10.00% | Dropout AE |
| Thase et al., 2007a | | 2 | 200 | | OFC | | 6-18mg titr. + 50mg | 8 | MADRS | 43.3 | 61.8 | 200 | 36.6 | 13.50% | AE discont | 26.00% | Dropout Any |
| Thase et al., 2007a | |  | 200 | | OFC | | 6-18mg titr. + 50mg |  |  | 45.3 | 70.4 | 200 | 44.3 | 13.50% | AE discont | 26.00% | Dropout Any |

| **Class/study reference** | **TRD code** | **N** | **Treatment** | **Dose** | **Duration (weeks)** | **Outcome measure** | **Avg age** | **% female** | **N analysed** | **% responded** | **Tolerability value** | **Tolerability measure (unit)** | **Acceptability Value** | **Acceptability measure (unit)** |
| --- | --- | --- | --- | --- | --- | --- | --- | --- | --- | --- | --- | --- | --- | --- |
| **NMDA modulators** |  |  |  |  |  |  |  |  |  |  |  |  |  |  |
| Daly et al., 2018 | 1 | 11/11/2012 | Esketamine | 30/60/90mg | 2 | MADRS | 42/43/50 | 46/82/50 | 08/11/2010 | 13/18/42 | 58/ 80/ 88% | Any TEAE | 37.5/ 0/ 0% | Dropout AE |
|  |  | 33 | Placebo | n/a |  |  | 44.4 | 54.5 | 32 | 6.3 | 55.00% | Any TEAE | 0.00% | Dropout AE |
| Fedgchin et al., 2019 | 2 | 117/ 116 | Esketamine | 60/90mg (2x week) | 4 | MADRS | 46.4/ 45.7 | 70.4/ 69.3 | 115/ 114 | 54.1/ 53.1 | 3.1/ 2.6 | TEAEs /ppt | 0.9/ 6.0% | Dropout AE |
|  |  | 113 | Placebo | n/a |  |  | 46.8 | 71.7 | 113 | 38.9 | 1.1 | TEAEs /ppt | 1.80% | Dropout AE |
| Heresco-Levy et al., 2006 | 0 | 22 | D-cycloserine | 250mg | 6 | HAMD-21 | 56.9 | 45.5 | 19 | nr | 5.30% | TEAE discont | 10.50% | Dropout Any |
|  |  | 22 | Placebo | n/a |  |  | 56.9 | 45.5 | 20 | nr | 25.00% | TEAE discont | 25.00% | Dropout Any |
| Heresco-Levy et al., 2013 | 2 | 13 | D-cycloserine | 250-1000mg titr. | 6 | HAMD-21 | 53 | 61.5 | 13 | 53.9 | 1.2 | TEAEs /ppt | 23.10% | Dropout Any |
|  |  | 13 | Placebo | 250-1000mg titr. |  |  | 53 | 38.5 | 13 | 15.4 | 1.2 | TEAEs /ppt | 7.7%% | Dropout Any |
| Husain et al., 2017 | 2 | 21 | Minocycline | 100-200mg titr. | 12 | HAMD-17 | 40 | 45 | 21 | 63 | 1 | AEs /ppt | 23.80% | Dropout Any |
|  |  | 20 | Placebo | n/a |  |  | 35 | 55 | 20 | 22 | 1 | AEs /ppt | 10.00% | Dropout Any |
| Ionescu et al., 2019 | 3 | 72 | Ketamine | 0.5mg/kg | 12 | HAMD-28 | 45.5 | 54 | 13 | 23.1 | 15.40% | TEAE discont | 5.60% | Dropout Any |
|  |  | 66 | Saline | n/a |  |  | 45.3 | 23 | 13 | 30.8 | 0.00% | TEAE discont | 3.10% | Dropout Any |
| Ochs-Ross et al., 2020 | 2 | 72 | Esketamine | 30-90mg titr. (2x week) | 4 | MADRS | 70.6 | 62.5 | 63 | 27 | 0.7 | TEAEs /ppt | 5.60% | Dropout Any |
|  |  | 66 | Placebo | n/a |  |  | 69.4 | 61.5 | 60 | 13.3 | 0.6 | TEAEs /ppt | 3.10% | Dropout Any |
| Popova et al., 2019 | 0 | 116 | Esketamine | 60-90mg titr. | 4 | MADRS | 44.9 | 65.8 | 114 | 69.3 | 2.9 | AEs /ppt | 7.00% | Dropout AE |
|  |  | 111 | Placebo | n/a |  |  | 46.4 | 57.8 | 109 | 52 | 0.9 | AEs /ppt | 0.90% | Dropout AE |
| Singh et al., 2016 | 1 | 18/ 17 | Ketamine | 0.5 mg/kg 2x/3x week | 4 | MADRS | 45.7/ 43.3 | 66.7/ 70.6 | 18/ 17 | 68.8/ 53.3 | 83.3/ 76.5% | Any AE | 11.1/ 5.9% | Dropout Any |
|  |  | 17/ 16 | Placebo | n/a |  |  | 40.3/ 46.1 | 75.0/ 56.3 | 16/ 16 | 15.4/ 6.3 | 56.3/ 50.0% | Any AE | 6.3/ 0.0% | Dropout Any |
| Su et al., 2017 | 3 | 23/24 | Ketamine | 0.2mg/kg / 0.5mg/kg | <1 | HAMD-17 | 45/48.5 | 73.9/ 87.5 | 23/ 24 | 39.1/ 45.8 | nr | nr | nr | nr |
|  |  | 24 | Placebo | n/a |  |  | 48.6 | 62.5 | 24 | 12.5 | nr | nr | nr | nr |

| **Class/study reference** | **TRD code** | **N** | **Treatment** | **Dose** | **Duration (weeks)** | **Outcome measure** | **Avg age** | **% female** | **N analysed** | **% responded** | **Tolerability value** | **Tolerability measure (unit)** | **Acceptability Value** | **Acceptability measure (unit)** |
| --- | --- | --- | --- | --- | --- | --- | --- | --- | --- | --- | --- | --- | --- | --- |
| **Mood stabilisers** |  |  |  |  |  |  |  |  |  |  |  |  |  |  |
| Barbee et al., 2011 | 2 | 48 | Lamotrigine | 25- 400mg titr. | 10 | MADRS | 44.6 | 68.8 | 48 | 33.3 | 87.50% | Any TEAE | nr | nr |
|  |  | 48 | Placebo | n/a |  |  | 45.8 | 68.8 | 48 | 33.3 | 87.50% | Any TEAE | nr | nr |
| Baumann et al., 1996 | 0 | 10 | Lithium | 800mg | 1 | HAMD-17 | 40 | 70 | 10 | 60 | 4.9 | TEAEs /ppt | 0.00% | Dropout Any |
|  |  | 14 | Placebo | n/a |  |  | 43 | 71.4 | 14 | 14.3 | 4.9 | TEAEs /ppt | 0.00% | Dropout Any |
| Girlanda et al., 2014 | 2 | 29 | Lithium | 444mg (mean) | 52 | QIDS-SR | 46 | 55 | 27 | nr | 0.4 | AEs /ppt | 7.40% | Dropout Any |
|  |  | 27 | TAU | n/a |  |  | 47 | 72 | 22 | nr | nr | nr | 22.70% | Dropout Any |
| Kok et al., 2007 | 0 | 15 | Lithium | Serum (range) 0.6-1.2mmol/L | 6 | MADRS | 73.6 | 73.3 | 15 | 46.7 | 4.3 | AEs /ppt | 0.00% | Dropout Any |
| Nierenberg et al., 2003 | 2 | 18 | Lithium | Serum (range/mean): (0.6-0.9 / 0.61 mmol/L) | 6 | HAMD-17 | 37.2 | 50 | 16 | 12.5 | nr | nr | nr | nr |
| Santos et al., 2008 | 2 | 17 | Lamotrigine | 200mg | 8 | MADRS | 26 | 82 | 16 | 26.7 | 1.9 | AEs /ppt | 17.60% | Dropout Any |
|  |  | 17 | Placebo | n/a |  |  | 29 | 65 | 16 | 35.7 | 1.6 | AEs /ppt | 23.50% | Dropout Any |
| Schindler et al., 2007 | 2 | 17 | Lithium | Serum (range/mean): 0.6-0.8 / 0.71mmol/L | 8 | HAMD-17 | 50.3 | 41.1 | 17 | 41.2 | 1.7 | TEAEs /ppt | 11.80% | Dropout Any |
|  |  | 17 | Lamotrigine | 25-250mg titr. |  |  | 45.1 | 52.9 | 17 | 52.9 | 0.9 | TEAEs /ppt | 11.80% | Dropout Any |
| Zusky et al., 1988 | 0 | 9 | Lithium | 300-900mg titr. | 2 to 3 | HAMD | 46.8 | 87.5 | 8 | 37.5 | nr | nr | 12.50% | Dropout TEAE |
|  |  | 9 | Placebo | n/a |  |  | 44.8 | 75 | 8 | 25 | nr | nr | 12.50% | Dropout TEAE |

| **Class/study reference** | **TRD code** | **N** | **Treatment** | **Dose** | **Duration (weeks)** | **Outcome measure** | **Avg age** | **% female** | **N analysed** | **% responded** | **Tolerability value** | **Tolerability measure (unit)** | **Acceptability Value** | **Acceptability measure (unit)** |
| --- | --- | --- | --- | --- | --- | --- | --- | --- | --- | --- | --- | --- | --- | --- |
| **Stimulants** |  |  |  |  |  |  |  |  |  |  |  |  |  |  |
| Cusin et al., 2013 | 0 | 30 | Pramipexole | 0.5-3mg titr. | 8 | MADRS | 47.3 | 53.3 | 30 | 40 | 7.1 | TEAEs /ppt | 13.30% | Dropout Any |
|  |  | 30 | Placebo | n/a |  |  | 45.5 | 60 | 30 | 26.7 | 6.9 | TEAEs /ppt | 13.30% | Dropout Any |
| DeBattista et al., 2003 | 0 | 69 | Modafinil | 100-400mg titr. | 6 | HAMD-21 | 45 | 67 | 68 | nr | 1.2 | TEAEs /ppt | 5.90% | Dropout Any |
|  |  | 67 | Placebo | n/a |  |  | 45 | 73 | 67 | nr | 0.9 | TEAEs /ppt | 4.50% | Dropout Any |
| Patkar et al., 2006 | 0 | 30 | Methylphenidate | 20-60mg titr. | 4 | HAMD-21 | 48.5 | 63 | 30 | 40 | 64% | Any TEAE | 6.70% | Dropout AE |
|  |  | 30 | Placebo | n/a |  |  | 48.5 | 60 | 30 | 23.3 | 58% | Any TEAE | 6.70% | Dropout AE |
| Ravindran et al., 2008 | 0 | 73 | Methylphenidate | 20-60mg titr. | 5 | MADRS | 45.6 | 64.4 | 72 | nr | 69.90% | Any AE | 8.20% | Dropout AE |
|  |  | 72 | Placebo | n/a |  |  | 41.9 | 65.3 | 72 | nr | 59.70% | Any AE | 0.00% | Dropout AE |
| Richards et al., 2016  (Study 1) | 0 | 202 | Lisdexamfetamine | 20-70mg titr. | 17 | MADRS | 42.2 | 64.2 | 201 | 40.8 | 65.20% | Any TEAE | 4.00% | Dropout TEAE |
|  |  | 202 | Placebo | n/a |  |  | 41.8 | 66.2 | 201 | 38.3 | 58.70% | Any TEAE | 3.50% | Dropout TEAE |
| Richards et al., 2016  (Study 2) |  | 212 | Lisdexamfetamine | 20-70mg titr. |  |  | 42 | 66.8 | 211 | 41.2 | 65.90% | Any TEAE | 0.90% | Dropout TEAE |
|  |  | 214 | Placebo | n/a |  |  | 42.6 | 67.1 | 213 | 37.1 | 50.70% | Any TEAE | 0.50% | Dropout TEAE |
| Richards et al., 2017 | 0 | 78x3 | Lisdexamfetamine | 10/10-30/10-50 mg titr. | 17 | MADRS | 39/43/44/42 | 69/68/68/66 | 77/76/78/80 | nr | 0.6/0.6/0.6/0.8 | AEs /ppt | 0/1.3/1.3/3.8% | Dropout TEAE |
|  |  | 78 | Placebo | n/a |  |  | 43.7 | 67.9 | 78 | nr | 0.4 | AEs /ppt | 0.00% | Dropout TEAE |

| **Class/study reference** | **TRD code** | **N** | **Treatment** | **Dose** | **Duration (weeks)** | **Outcome measure** | **Avg age** | **% female** | **N analysed** | **% responded** | **Tolerability value** | **Tolerability measure (unit)** | **Acceptability Value** | **Acceptability measure (unit)** |
| --- | --- | --- | --- | --- | --- | --- | --- | --- | --- | --- | --- | --- | --- | --- |
| **Hormones** |  |  |  |  |  |  |  |  |  |  |  |  |  |  |
| Gitlin et al., 1987 | 0 | 7 | T3 | 25ug | 2 | HAMD | 41 | nr | 7 | nr | nr | nr | nr | nr |
|  |  | 9 | Placebo | n/a |  |  | 41 | nr | 9 | nr | nr | nr | nr | nr |
| Joffe & Singer., 1990 | 0 | 21 | T4 | 150ug | 3 | HAMD-17 | 34.5 | 71.4 | 21 | 19 | nr | nr | 0.00% | Dropout Any |
|  |  | 19 | T3 | 37.5ug |  |  | 34.5 | 52.6 | 17 | 53 | nr | nr | 11.80% | Dropout Any |
| Pope et al., 2003 | 0 | 12 | Testosterone Gel | 7.5-10g titr. | 8 | HAMD | 48.9 | 0 | 11 | nr | 9.10% | TEAE discont | 18.20% | Dropout Any |
|  |  | 10 | Placebo | n/a |  |  | 49.5 | 0 | 10 | nr | 0.00% | TEAE discont | 10.00% | Dropout Any |
| Pope et al., 2010 | 0 | 50 | Testosterone Gel | 2.5-10g titr. | 6 | HAMD-21 | 50.6 | 0 | 49 | 26.5 | 0.3 | TEAEs /ppt | 18.40% | Dropout Any |
|  |  | 50 | Placebo | 5g |  |  | 49.9 | 0 | 46 | 13 | 0 | TEAEs /ppt | 26.10% | Dropout Any |
| Seidman et al., 2005 | 1 | 13 | Testosterone enanthate | 200-600mg titr. | 6 | HAMD -24 | 46.4 | 0 | 13 | 53.8 | 0.00% | Any AE | 0.00% | Dropout Any |
|  |  | 13 | Placebo | n/a |  |  | 46.4 | 0 | 13 | 23.1 | 0.00% | Any AE | 23.10% | Dropout Any |

| **Class/study reference** | **TRD code** | **N** | **Treatment** | **Dose** | **Duration (weeks)** | **Outcome measure** | **Avg age** | **% female** | **N analysed** | **% responded** | **Tolerability value** | **Tolerability measure (unit)** | **Acceptability Value** | **Acceptability measure (unit)** |
| --- | --- | --- | --- | --- | --- | --- | --- | --- | --- | --- | --- | --- | --- | --- |
| **Opioids** |  |  |  |  |  |  |  |  |  |  |  |  |  |  |
| Fava et al., 2016 | 0 | 23/ 22 | Buprenorphine | 2mg (2mg)/ 8mg (8mg) | 10 | HAMD-17 | 45.2/45.8 | 70.8/ 57.9 | 22/ 21 | 47.0/ 36.0 | 80.9/ 90.2% | Any TEAE | 31.8/ 31.6% | Dropout Any |
|  |  | 98 | Placebo | n/a |  |  | 46.6 | 69.4 | 97 | 23.7 | 65.60% | Any TEAE | 10.30% | Dropout Any |
| Lin et al., 2019 | 0 | 20 | Buprenorphine | 0.2-1.2mg titr. | 8 | MADRS | 65 | 37.5 | 16 | nr | -1 | TEAE increase | 25.00% | Dropout Any |
|  |  | 11 | Placebo | n/a |  |  | 66 | 27.3 | 11 | nr | -3 | TEAE increase | 0.00% | Dropout Any |
| Zajecka et al., 2019 | 0 | 149 | Buprenorphine | 2mg (2mg) | 6 | MADRS-10 | 47.4 | 59.9 | 131 | 16.9 | 42.90% | Any AE | 10.70% | Dropout Any |
|  |  | 148 | Placebo | n/a | 10 |  | 48.1 | 63.5 | 138 | 14.4 | 34.50% | Any AE | 8.70% | Dropout Any |

| **Class/study reference** | **TRD code** | **N** | **Treatment** | **Dose** | **Duration (weeks)** | **Outcome measure** | **Avg age** | **% female** | **N analysed** | **% responded** | **Tolerability value** | **Tolerability measure (unit)** | **Acceptability Value** | **Acceptability measure (unit)** |
| --- | --- | --- | --- | --- | --- | --- | --- | --- | --- | --- | --- | --- | --- | --- |
| **Vitamins** |  |  |  |  |  |  |  |  |  |  |  |  |  |  |
| Papakostas et al., 2012° | 0 | 35 | L-methylfolate | 7.5-15mg | 8.6 | HAMD-17 | 47.9 | 69.5 | 35 | 17.1 | nr | nr | 8.30% | Dropout Any |
|  |  | 33 | Placebo | n/a |  |  | 47.9 | 69.5 | 33 | 9 | 1 | TEAEs /ppt | 12.50% | Dropout Any |
| Papakostas et al., 2012° |  | 18 | L-methylfolate | 15mg |  |  | 48.4 | 70.6 | 18 | 27.8 | 2.3 | TEAEs /ppt | 16.70% | Dropout Any |
|  |  | 21 | Placebo | n/a |  |  | 48.4 | 70.6 | 21 | 9.5 | 2.6 | TEAEs /ppt | 14.30% | Dropout Any |

| **Class/study reference** | **TRD code** | **N** | **Treatment** | **Dose** | **Duration (weeks)** | **Outcome measure** | **Avg age** | **% female** | **N analysed** | **% responded** | **Tolerability value** | **Tolerability measure (unit)** | **Acceptability Value** | **Acceptability measure (unit)** |
| --- | --- | --- | --- | --- | --- | --- | --- | --- | --- | --- | --- | --- | --- | --- |
| **Other treatment classes** |  |  |  |  |  |  |  |  |  |  |  |  |  |  |
| Kessler et al., 2018 | 0 | 241 | Mirtazapine | 30mg | 12 | BDI -II | 50.4 | 69.7 | 214 | 43.9 | 56.50% | Any AE | 8.70% | Dropout Any |
|  |  | 239 | Placebo | n/a |  |  | 49.9 | 68.6 | 217 | 35.9 | 32.70% | Any AE | 6.30% | Dropout Any |
| Lin et al., 2020 | 1 | 48 | Agomelatine | 50mg | 3 to 6 | HAMD-17 | 49.4 | 63.4 | 41 | 92.7 | 1.6 | AEs /ppt | nr | nr |
|  |  | 49 | Placebo | n/a |  |  | 54 | 63.4 | 41 | 90.2 | 1.4 | AEs /ppt | nr | nr |
| McAllister-Williams et al., 2016 | 2 | 83 | Metyrapone | 1000mg | 3 | MADRS | 47.6 | 56.6 | 69 | 20.3 | 1.9 | AEs /ppt | 20.30% | Dropout Any |
|  |  | 82 | Placebo | n/a |  |  | 45.2 | 63.4 | 74 | 21.6 | 1.3 | AEs /ppt | 10.80% | Dropout Any |
| Medhus et al., 1994 | 0 | 18 | Mianserin | 30-60mg titr. | 3 | MADRS | 47.5 | 61.1 | 18 | nr | nr | nr | 11.10% | Dropout Any |
|  |  | 19 | Placebo | nr |  |  | 47 | 68.4 | 19 | nr | nr | nr | 5.30% | Dropout Any |
| Appelberg et al., 2001 | 0 | 51 | Buspirone | 10-30mg titr. | 6 | MADRS | 44 | 63 | 51 | 33 | nr | nr | 11.80% | Dropout Any |
|  |  | 51 | Placebo | n/a |  |  | 44 | 63 | 51 | 31.3 | nr | nr | 19.60% | Dropout Any |
| George et al., 2008 | 0 | 11 | Mecamylamine | 5mg - 10mg | 8 | HAMD-17 | 49.6 | 81.8 | 11 | 45.4 | 2.2 | AEs /ppt | 6.5/8 | Retention (weeks) |
|  |  | 10 | Placebo | n/a |  |  | 48.3 | 80 | 10 | 10 | 0.7 | AEs /ppt | 7.6/8 | Retention (weeks) |
| Gulrez et al., 2012 | 0 | 30 | Bupropion | 300mg | 4 | MADRS | 39.2 | 54 | 30 | nr | nr | nr | nr | nr |
|  |  | 30 | Placebo | n/a |  |  | 43.2 | 50 | 30 | nr | nr | nr | nr | nr |
| Matthew et al., 2017 ° | 0 | 25 | Riluzole | 100mg | 8 | MADRS | 44.5 | 36 | 25 | 24 | Symptom specific* | SAFTEE-SI | 16.00% | Dropout Any |
|  |  | 40 | Placebo | n/a |  |  | 46.3 | 52.5 | 40 | 25 | Symptom specific** | SAFTEE-SI | 12.50% | Dropout Any |
| Möller et al., 2015 | 2 | 160/160/160 | Dexmecamylamine | 1/4/ 8mg | 8 | HAMD-17 | 41/42/42 | 53/57/58 | 155/156/ 152 | 44/40/49 | 63.1/ 62.7/ 77.8% | Any AE | 5.7/ 7.5/ 13.2% | Dropout AE |
|  |  | 161 | Placebo | n/a |  |  | 43.2 | 59.6 | 157 | 42.7 | 59.40% | Any AE | 1.70% | Dropout AE |
| Möller et al., 2015 |  | 174/174/174 | Dexmecamylamine | 0.2/2/8mg |  |  | 46/45/46 | 73/72/67 | 173/174/174 | 49/51/41 | 46.0/ 56.3/ 64.9% | Any AE | 1.9/ 5.6/ 13.1% | Dropout AE |
|  |  | 174 | Placebo | n/a |  |  | 45.8 | 74.1 | 174 | 54 | 52.90% | Any AE | 4.30% | Dropout AE |
| Papakostas et al., 2010 | 0 | 39 | S-adenosyl methionine | 800-1600mg | 6 | HAMD-17 | nr | 53.8 | 39 | 51.3 | 0.9 | AEs /ppt | 5.10% | Dropout intolerance |
|  |  | 34 | Placebo | n/a |  |  | nr | 67.6 | 34 | 20.6 | 0.7 | AEs /ppt | 8.80% | Dropout intolerance |
| Perez et al., 1999 | 0 | 40 | Pindolol | 7.5mg | 1.5 | HAMD-17 | 46.2 | 75 | 40 | 12.5 | 0.2 | TEAEs /ppt | nr | nr |
|  |  | 40 | Placebo | n/a |  |  | 48 | 67.5 | 40 | 12.5 | 0.1 | TEAEs /ppt | nr | nr |
| Perry et al., 2004 | 0 | 22 | Pindolol | 7.5mg | 6 | HAMD-25 | 49 | 76.2 | 21 | 19 | nr | nr | 9.50% | Dropout Any |
|  |  | 20 | Placebo | n/a |  |  | 43 | 70.6 | 17 | 29.4 | nr | nr | 23.50% | Dropout Any |
| Targum et al., 2018 | 0 | 118 | S-adenosyl methionine | 800mg | 6 | HAMD-17 | 48.3 | 66.1 | 117 | nr | 44.10% | Any TEAE | 0.90% | Dropout Any |
|  |  | 116 | Placebo | n/a |  |  | 46.1 | 68.1 | 110 | nr | 37.10% | Any TEAE | 0.90% | Dropout Any |

CBT; cognitive behavioural therapy, iCBT; internet / smartphone / web based CBT, TAU; treatment as usual, OFC; olanzapine-fluoxetine combination. Titr. = titrated. MADRS = Montgomery-Asberg Depression Rating Scale; HAMD = Hamilton Depression Scale; QIDS = Quick Inventory of Depressive Symptomology; BDI = Beck’s Depression Inventory; PHQ = Patient Health Questionnaire, nr = not recorded; AE = adverse event; TEAE = treatment emergent adverse event; SAS = Simpson and Angus scale; SAE = serious adverse event; BAS = Barnes' Akathisia Rating scale.

TRD Code definitions: 0 = participants with a minimum of one failed treatment could be classed as TRD; 1 = two failed treatments with either or both lasting less than six weeks; 2 = two antidepressant medications in the current episode for a minimum of six weeks (this could include one prospective trial as part of the study design); 3 = three or more failed trials, including a prospective trial as part of the study design.

**Supplementary Table 2: risk of bias ratings from included studies**

| **Study Reference** | **Potential sources of bias** | | | | | | | | | **Overall RoB Judgement** |
| --- | --- | --- | --- | --- | --- | --- | --- | --- | --- | --- |
|  | **A** | **B** | **C** | **D** | **E** | **F** | **G** | **H** | **I** |  |
| Adli et al., 2017 | + | + | ? | - | + | - | + | + | + | Moderate |
| Astellas Pharma. | + | ? | ? | ? | ? | + | + | + | – | High |
| Appelberg et al., 2001 | + | ? | ? | + | + | + | + | + | ? | Moderate |
| Barbee et al., 2011 | + | + | + | + | – | + | + | + | – | Moderate |
| Bauer et al., 2009 | + | ? | ? | + | + | + | + | + | + | Low |
| Bauer et al., 2013 | + | + | + | – | + | + | + | + | – | Moderate |
| Bauer et al., 2018 | + | + | + | + | ? | + | + | + | – | Low |
| Baumann et al., 1996 | + | ? | ? | + | + | + | + | + | ? | Moderate |
| Berman et al., 2007 | + | ? | ? | + | + | + | + | – | – | Moderate |
| Berman et al., 2009 | + | ? | ? | + | + | + | + | – | – | Moderate |
| Chaput et al., 2008 | + | ? | ? | + | + | + | + | + | + | Low |
| Cheon et al., 2017 | + | ? | – | – | + | + | + | + | + | Moderate |
| Corya et al., 2006 | + | ? | – | + | + | + | + | + | – | Moderate |
| Cusin et al., 2013 | + | ? | ? | + | + | + | + | + | + | Low |
| Daly et al., 2018 | + | + | + | + | + | + | + | + | – | Low |
| DeBattista et al., 2003 | + | ? | ? | + | + | – | + | + | – | Moderate |
| Domany et al., 2019 | + | ? | ? | ? | – | + | + | + | + | Moderate |
| Doree et al., 2007 | + | ? | ? | – | + | + | + | + | + | Moderate |
| Dunner et al., 2007 | + | ? | ? | ? | + | + | + | + | + | Moderate |
| Durgam et al., 2016 | + | + | ? | + | + | + | + | + | – | Low |
| Eisendrath et al., 2016 | + | + | + | + | + | + | + | – | + | Low |
| El-Khalili et al., 2010 | + | + | ? | + | + | + | + | + | – | Low |
| Fang et al., 2011 | + | + | + | + | ? | + | + | + | + | Low |
| Fava et al., 1994 | + | ? | ? | + | ? | ? | + | + | + | Moderate |
| Fava et al., 2002 | + | ? | – | + | + | + | + | + | + | Low |
| Fava et al., 2012 | + | ? | ? | + | + | + | + | + | – | Moderate |
| Fava et al., 2016 | + | ? | ? | + | + | + | + | + | – | Moderate |
| Fava et al., 2018 | + | + | + | + | ? | ? | + | + | – | Moderate |
| Fedgchin et al, 2019 | + | + | ? | + | + | – | + | + | – | Moderate |
| Fonagy et al., 2015 | + | + | ? | + | – | + | + | + | + | Low |
| Fornaro et al., 2014 | + | ? | ? | + | ? | + | + | + | + | Moderate |
| Franco-Chaves et al., 2013 | + | + | ? | + | + | + | + | + | + | Low |
| Gamble et al., 2018 | + | + | + | + | – | + | + | + | + | Low |
| George et al., 2008 | + | ? | ? | + | + | + | + | + | – | Moderate |
| Girlanda et al., 2014 | + | + | + | – | + | + | + | + | + | Low |
| Han et al., 2015 | + | + | + | + | + | + | + | + | + | Low |
| Harley et al., 2008 | + | + | ? | + | + | + | + | – | ? | Moderate |
| Hauksson et al., 2017 | + | – | ? | ? | + | + | + | + | + | Moderate |
| Heresco-Levy et al, 2006 | + | ? | ? | + | + | – | + | + | + | Moderate |
| Heresco-Levy et al., 2013 | + | + | ? | + | + | + | + | + | – | Low |
| Hobart et al., 2018a | + | + | + | + | + | + | + | + | – | Low |
| Hobart et al., 2018b | + | + | + | + | + | + | + | + | – | Low |
| Horikoshi et al., 2019 | + | ? | ? | ? | + | + | + | + | + | Moderate |
| Husain et al., 2017 | + | + | + | + | + | + | + | + | + | Low |
| Ionescu et al., 2010 | + | ? | ? | + | + | + | + | + | – | Moderate |
| Ionescu et al., 2019 | + | + | ? | + | + | + | + | + | – | Low |
| Joffe & Singer., 1990 | + | + | + | + | ? | + | + | – | + | Low |
| Joffe et al., 1993 | + | ? | ? | – | + | + | + | – | + | Moderate |
| Joffe et al., 2006 | + | ? | ? | + | + | + | + | + | + | Low |
| Kamijima et al., 2013 | + | ? | ? | + | + | ? | + | + | – | Moderate |
| Kamijima et al., 2018 | + | ? | + | + | + | ? | + | + | – | Moderate |
| Kato et al., 2017 | + | ? | – | – | + | + | + | + | + | Moderate |
| Kessler et al., 2018 | + | + | + | + | – | + | + | + | + | Low |
| Kocsis et al., 2012 | + | + | ? | ? | + | + | + | – | + | Moderate |
| Kok et al., 2007 | + | + | + | – | + | + | + | + | + | Low |
| Lenze et al., 2015 | + | + | + | + | + | + | + | + | + | Low |
| Licht et al., 2002 | + | + | + | + | + | + | + | + | – | Low |
| Lin et al., 2019 | + | – | ? | + | + | + | + | + | – | Moderate |
| Lin et al., 2020 | + | + | + | + | + | + | + | + | + | Low |
| Maes et al., 1996 | + | ? | ? | + | + | + | + | + | ? | Moderate |
| Mahmoud et al., 2007 | + | + | + | + | + | + | + | + | – | Low |
| Mantani et al., 2017 | + | + | + | + | + | + | + | + | – | Low |
| Marcus et al., 2008 | + | ? | – | + | + | + | + | – | – | Moderate |
| Mathew et al., 2017 | + | + | + | + | + | + | + | + | – | Low |
| McAllister-Williams et al., 2016 | + | + | + | + | + | ? | + | – | + | Low |
| McGrath et al., 2006 | + | ? | ? | + | – | + | + | + | + | Moderate |
| McIntyre et al., 2007 | + | ? | ? | + | + | + | + | + | – | Moderate |
| Medhus et al., 1994 | + | ? | ? | + | + | + | + | + | ? | Moderate |
| Mohamed et al., 2017 | + | + | ? | – | + | + | + | + | + | Low |
| Möller et al., 2015 (two studies) | + | + | + | + | + | – | + | + | – | Moderate |
| Nakagawa et al., 2017 | + | + | + | + | + | + | + | + | + | Low |
| Nakao et al., 2018 | + | + | + | + | + | + | + | + | + | Low |
| Navarro et al., 2019 | + | + | ? | – | + | + | + | + | + | Low |
| Nierenberg et al., 2003 | + | ? | ? | + | + | + | + | + | + | Low |
| Nierenberg et al., 2006 | + | ? | – | – | – | – | + | + | + | High |
| Ochs-Ross et al., 2020 | + | + | ? | + | + | – | + | + | – | Moderate |
| Papakostas et al., 2010 | + | ? | ? | + | + | + | + | + | + | Low |
| Papakostas et al., 2012 (two studies) | + | ? | ? | + | ? | + | + | + | – | Moderate |
| Papakostas et al., 2015 | + | + | + | + | + | + | + | + | + | Low |
| Patkar et al., 2006 | + | ? | ? | + | ? | – | + | + | – | High |
| Perez et al., 1999 | + | + | + | + | + | + | + | + | + | Low |
| Perry et al., 2004 | + | ? | ? | + | + | + | + | + | + | Low |
| Pope et al., 2003 | + | ? | – | + | + | + | + | + | + | Low |
| Pope et al., 2010 | + | + | + | + | + | – | + | + | + | Low |
| Popova et al., 2019 | + | + | + | + | + | + | + | + | – | Low |
| Ravindran et al., 2008 | + | ? | ? | + | + | – | + | + | – | Moderate |
| Reeves et al., 2008 | + | ? | ? | + | – | + | + | + | + | Moderate |
| Richards et al., 2016 (two studies) | + | + | + | + | + | – | + | + | ? | Low |
| Richards et al., 2017 | + | + | + | + | + | – | + | + | – | Moderate |
| Santos et al., 2008 | + | + | + | + | + | + | + | – | + | Low |
| Schindler et al., 2007 | + | + | – | – | – | – | – | + | ? | High |
| Seidman et al., 2005 | + | + | + | + | + | + | + | + | + | Low |
| Shelton et al., 2001 | + | ? | ? | + | + | + | ? | + | – | Moderate |
| Shelton et al., 2005 | + | + | ? | + | + | + | + | + | – | Low |
| Singh et al., 2016 | + | + | + | + | + | + | + | + | – | Low |
| Souza et al., 2016 | + | + | + | + | + | + | + | + | ? | Low |
| Stabl et al., 1995 | + | ? | ? | + | + | + | + | + | – | Moderate |
| Su et al., 2017 | + | ? | ? | + | + | + | + | + | + | Low |
| Targum et al., 2018 | + | ? | ? | + | – | + | + | + | – | Moderate |
| Thase et al., 2007a | + | + | ? | + | + | + | + | + | + | Low |
| Thase et al., 2007b (two studies) | + | ? | ? | + | + | + | + | + | + | Low |
| Thase et al., 2015a | + | + | + | + | + | + | + | + | – | Low |
| Thase et al., 2015b | + | + | + | + | + | + | + | + | – | Low |
| Town et al., 2017 | + | + | + | + | + | ? | + | + | + | Low |
| Trivedi et al., 2006 | + | ? | ? | – | + | + | + | + | + | Moderate |
| Wiles et al., 2008 | + | + | + | – | – | + | – | + | + | Moderate |
| Wiles et al., 2013 / 2014 | + | + | + | – | – | + | + | + | + | Moderate |
| Yoshimura et al., 2012 | + | ? | ? | – | + | + | + | – | ? | High |
| Yoshimura et al., 2014 | + | ? | ? | ? | + | + | + | ? | + | Moderate |
| Zajecka et al., 2019 | + | ? | + | + | + | ? | + | + | – | Moderate |
| Zusky et al., 1988 | + | ? | – | + | + | + | – | + | + | Moderate |

**Supplementary Table 2a.** Evaluations of the 115 included studies for potential sources of bias according to the SIGN tool (Scottish Intercollegiate Guidelines Network, 2012).

A. Question appropriate and clearly focused; B. Allocation sequence randomly generated; C. Allocation adequately concealed; D. Knowledge of allocation adequately prevented (blinding); E. Group comparability at baseline ensured; F. Differences among multiple sites adequately addressed; G. Selective outcome reporting avoided; H. Intention-to-treat analysis applied; I. Allegiance effect minimised.

**+**, low risk / **–**, high risk / **?**, unclear risk

Overall risk was judged as follows:

*Low risk = <2 criteria rated high RoB (or <2 if at least one is rated unclear RoB)*

*Moderate risk = 2-4 criteria rated high or unclear RoB*

*High risk = >4 criteria rated high or unclear RoB*

| **Treatment Modality** | **Random sequence generation** | **Allocation concealment** | **Blinding** | **Comparable groups** | **Equal treatment** | **Standardised outcomes** | **ITT analysis** | **Allegiance** | **Overall** |
| --- | --- | --- | --- | --- | --- | --- | --- | --- | --- |
| **ANTIPSYCHOTICS** | 0.288 | 0.327 | 0.135 | 0.077 | 0.096 | 0 | 0.154 | 0.712 | 1.54 |
| **COMBINATION T** | 0.35 | 0.5 | 0 | 0.15 | 0 | 0.05 | 0 | 0.5 | 1.3 |
| **HORMONES** | 0.25 | 0.25 | 0 | 0.167 | 0.333 | 0 | 0.25 | 0.167 | 1.5 |
| **MOOD STABILISER** | 0.188 | 0.375 | 0.375 | 0.25 | 0.125 | 0.25 | 0.125 | 0.25 | 1.63 |
| **MULTIPLE TREATMENTS** | 0.231 | 0.365 | 0.442 | 0.173 | 0.115 | 0.038 | 0.096 | 0.115 | 1.73 |
| **NMDA MODULATORS** | 0.1 | 0.25 | 0 | 0.1 | 0.3 | 0 | 0 | 0.6 | 1.5 |
| **OPIOIDS** | 0.75 | 0.5 | 0 | 0 | 0 | 0 | 0 | 1 | 1.5 |
| **OTHER MEDICATIONS** | 0.269 | 0.308 | 0.077 | 0.231 | 0.115 | 0 | 0.077 | 0.385 | 1.38 |
| **PSYCHOLOGICAL** | 0 | 0.333 | 0 | 0.333 | 0 | 0 | 0.667 | 0.167 | 1.33 |
| **STIMULANTS** | 0.333 | 0.333 | 0 | 0.083 | 0.833 | 0 | 0 | 0.75 | 1.5 |
| **VITAMINS** | 0.5 | 0.5 | 0 | 0.5 | 0 | 0 | 0 | 1 | 1 |
| **ALL TREATMENTS** | **0.257** | **0.347** | **0.171** | **0.153** | **0.162** | **0.032** | **0.108** | **0.437** | **1.53** |

**Supplementary Table 2b.**

**Supplementary Table 2b.** Risk of bias ratings stratified by treatment class. Scores range from 0-1, signifying low-high risk of bias. NB. Criterion 1 (appropriately focussed question) is omitted from the table due to homogeneity.

**Supplementary Table 3.** Results of combination study effects

| **Combination studies (all k = 1)** | | *n* | *ES* | *SE* | *95% CI* |
| --- | --- | --- | --- | --- | --- |
|  | Escitalopram & Pramipexole | 13 | 0.39 | 0.29 | -0.18 – 0.95 |
|  | Duloxetine & Bupropion | 24 | 1.10 | 0.71 | -0.29 – 2.50 |
|  | Duloxetine & Placebo | 22 | 0.32 | 0.22 | -0.10 – 0.75 |
|  | Venlafaxine & Mirtazapine | 58 | 0.74 | 0.15 | 0.45 – 1.03 |
|  | CBT & Quetiapine | 11 | 1.40 | 0.42 | 0.57 – 2.23 |
|  | CBT & Placebo | 11 | 0.27 | 0.31 | -0.33 – 0.87 |
|  | Thyroid & Lithium | 9 | 0.80 | 0.38 | 0.05 – 1.55 |
|  | Ketamine & ECT | 12 | 5.22 | 1.10 | 3.05 – 7.38 |
|  | CBASP & med optimisation | 174 | 0.99 | 0.09 | 0.81 – 1.18 |
|  | Active psy control & med optimisation | 168 | 0.80 | 0.09 | 0.62 – 0.97 |
|  | CBT & medication switch | 81 | 0.75 | 0.13 | 0.51 – 1.00 |
|  | TAU & medication switch | 83 | 0.25 | 0.11 | 0.03 – 0.47 |

NB OFC studies are included in the main tables as this is an accepted and more commonly studied adjunctive medication strategy

**Supplementary Table 4**

| **Treatment** |  | **k** | **n** | **ES** | **SE** | **95% CI** | ***I*^2^** |
| --- | --- | --- | --- | --- | --- | --- | --- |
| Aripiprazole | 1FT | 8 | 1447 | 1.26 | 0.15 | 0.97 – 1.55 | 91% |
|  | 2FT | 4 | 524 | 1.30 | 0.05 | 1.20 – 1.40 | 0% |
| Cariprazine | 1FT | 1 | 544 | 1.17 | 0.06 | 1.06 – 1.28 | n/a |
|  | 2FT | 1 | 149 | 0.98 | 0.10 | 0.78 – 1.17 | n/a |
| Olanzapine ^a^ | 1FT | 2 | 210 | 1.25 | 0.09 | 1.07 – 1.43 | 0% |
|  | 2FT | 1 | 10 | 0.98 | 0.38 | 0.22 – 1.73 | n/a |
| Quetiapine | 1FT | 4 | 655 | 1.38 | 0.11 | 1.17 – 1.58 | 55% |
|  | 2FT | 2 | 329 | 0.98 | 0.16 | 0.68 – 1.29 | 79% |
| Risperidone | 1FT | 4 | 255 | 1.50 | 0.20 | 1.10 – 1.89 | 73% |
|  | 2FT | 1 | 45 | 1.14 | 0.19 | 0.78 – 1.53 | n/a |
| Ziprasidone ^a^ | 1FT | 1 | 71 | 1.00 | 0.14 | 0.72 – 1.29 | n/a |
|  | 2FT | 1 | 41 | 0.66 | 0.18 | 0.31 – 1.01 | n/a |
| Lithium ^a^ | 1FT | 9 | 152 | 1.34 | 0.17 | 1.00 – 1.67 | 43% |
|  | 2FT | 4 | 278 | 0.93 | 0.07 | 0.79 – 1.07 | 0% |
| Thyroid | 1FT | 3 | 55 | 1.33 | 0.38 | 0.58 – 2.08 | 74% |
|  | 2FT | 1 | 48 | 1.15 | 0.19 | 0.79 – 1.52 | n/a |
| d-cycloserine ^a^ | 1FT | 1 | 19 | 1.48 | 0.46 | 0.58 – 2.37 | n/a |
|  | 2FT | 1 | 10 | 1.37 | 0.32 | 0.74 – 1.99 | n/a |
| Ketamine | 1FT | 5 | 495 | 1.55 | 0.19 | 1.26 – 1.52 | 85% |
|  | 2FT | 3 | 82 | 1.45 | 0.11 | 1.23 – 1.67 | 0% |
| Buspirone | 1FT | 2 | 337 | 0.84 | 0.12 | 0.61 – 1.07 | 46% |
|  | 2FT | 1 | 46 | 1.57 | 0.22 | 1.14 – 2.02 | n/a |
| Mecamylamine ^a^ | 1FT | 1 | 11 | 1.86 | 0.50 | 0.88 – 2.83 | n/a |
|  | 2FT | 1 | 490 | 1.13 | 0.18 | 0.78 – 1.49 | n/a |
| CBT/CT | 1FT | 5 | 203 | 1.54 | 0.28 | 1.00 – 2.09 | 89% |
|  | 2FT | 1 | 142 | 1.75 | 0.20 | 1.36 – 2.14 | n/a |
| Other placebo | 1FT | 8 | 404 | 0.73 | 0.12 | 0.51 – 0.96 | 72% |
|  | 2FT | 2 | 37 | 0.69 | 0.13 | 0.43 – 0.94 | 0% |
| Pill placebo | 1FT | 38 | 3029 | 0.95 | 0.06 | 0.83 – 1.08 | 86% |
|  | 2FT | 20 | 2577 | 0.81 | 0.05 | 0.71 – 0.90 | 67% |
| TAU | 1FT | 7 | 345 | 0.81 | 0.15 | 0.52 – 1.10 | 70% |
|  | 2FT | 3 | 109 | 0.86 | 0.26 | 0.35 – 1.36 | 78% |

**Supplementary Table 4.** Outlines the impact of degree of treatment-resistance on ES for augmentation modalities.

1FT: one failed adequate treatment.

2FT: failure of two adequate treatment trials.

Blue text indicates subgroups where heterogeneity had reduced from significant (disregarding subgroup) to non-significant according to our previous criteria of *I*^2^ < 60%.

**Supplementary Table 5**

| **Treatment** |  | **k** | **n** | **ES** | **SE** | **95% CI** | ***I*^2^** |
| --- | --- | --- | --- | --- | --- | --- | --- |
| Mirtazapine ^a^ | ST | 1 | 10 | 1.35 | 0.49 | 0.40 – 2.30 | n/a |
|  | AD | 1 | 214 | 1.19 | 0.09 | 1.01 – 1.36 | n/a |
| Bupropion | ST | 1 | 30 | 1.59 | 0.28 | 1.05 – 2.13 | n/a |
|  | AD | 3 | 831 | 1.07 | 0.44 | 0.20 – 1.93 | 99% |
| Aripiprazole | ST | 1 | 10 | 1.15 | 0.03 | 1.23 – 1.35 | n/a |
|  | AD | 11 | 1961 | 1.28 | 0.09 | 1.10 – 1.46 | 88% |
| Olanzapine ^a^ | ST | 1 | 10 | 1.51 | 0.46 | 0.61 – 2.42 | n/a |
|  | AD | 2 | 210 | 1.22 | 0.09 | 1.04 – 1.40 | 0% |
| Risperidone | ST | 1 | 64 | 1.17 | 0.16 | 0.85 – 1.48 | n/a |
|  | AD | 4 | 236 | 1.50 | 0.20 | 1.11 – 1.89 | 70% |
| Lithium ^a^ | ST | 7 | 77 | 1.02 | 0.19 | 0.65 – 1.38 | 36% |
|  | AD | 5 | 326 | 1.35 | 0.21 | 0.95 – 1.75 | 70% |
|  | LT | 1 | 27 | 0.86 | 0.23 | 0.41 – 1.29 | n/a |
| Thyroid | ST | 3 | 55 | 1.33 | 0.38 | 0.58 – 2.08 | 74% |
|  | AD | 1 | 48 | 1.15 | 0.19 | 0.79 – 1.52 | n/a |
| Pindolol ^a^ | ST | 2 | 61 | 0.90 | 0.28 | 0.36 – 1.44 | 44% |
|  | AD | 1 | 11 | 0.83 | 0.25 | 0.34 – 1.33 | n/a |
| Pill placebo | ST | 13 | 467 | 0.85 | 0.11 | 0.64 – 1.07 | 72% |
|  | AD | 44 | 5139 | 0.90 | 0.05 | 0.81 – 0.99 | 84% |
| TAU | AD | 8 | 381 | 0.86 | 0.14 | 0.59 – 1.13 | 71% |
|  | LT | 2 | 73 | 0.71 | 0.32 | 0.08 – 1.34 | 77% |

**Supplementary Table 5.** subgroup analysis examining the effect of duration on ESs.

ST: short-term (< 6 weeks, with the exception of rapid acting (es)ketamine interventions)

AD: adequate duration (6-12 weeks)

LT: long-term (>12 weeks)

Blue text indicates subgroups where heterogeneity had reduced from significant (disregarding subgroup) to non-significant according to our previous criteria of *I*^2^ < 60%.

**Supplementary reference list**

Adli M, Wiethoff K, Baghai TC, Fisher R, Seemüller F, et al. (2017) How Effective Is Algorithm-Guided Treatment for Depressed Inpatients? Results from the Randomized Controlled Multicenter German Algorithm Project 3 Trial. International Journal of Neuropsychopharmacology 20(9): 721-730.

Appelberg BG, Syvälahti EK, Koskinen TE, Mehtonen OP, Muhonen TT, et al. (2001) Patients With Severe Depression May Benefit From Buspirone Augmentation of Selective Serotonin Reuptake Inhibitors: Results From a Placebo-Controlled, Randomized, Double-Blind, Placebo Wash-In Study. Journal of Clinical Psychiatry 62: 448-452.

Astellas Pharmaceutical Industries. (n.d.). Study to evaluate the effect and safety of quetiapine extended release (XR) (FK949E) in major depressive disorder. ClinicalTrials.gov.

Barbee JG, Thompson TR, Jamhour NJ, Stewart JW, Conrad EJ, et al. (2011) A Double-Blind Placebo-Controlled Trial of Lamotrigine as an Antidepressant Augmentation Agent in Treatment-Refractory Unipolar Depression. Journal of Clinical Psychiatry 72(10): 1405-1412.

Bauer M, Dell'Osso L, Kasper S, Pitchot W, Vansvik ED, et al. (2013) Extended-release quetiapine fumarate (quetiapine XR) monotherapy and quetiapine XR or lithium as add-on to antidepressants in patients with treatment-resistant major depressive disorder. *Journal of Affective Disorders* 151: 209-219.

Bauer M, Hefting N, Lindsten A, Josiassen MK and Hobart M (2018) A randomised, placebo-controlled 24-week study evaluating adjunctive brexpiprazole in patients with major depressive disorder. *Acta Neuropsychiatrica* Epub ahead of print doi: 10.1017/ neu.2018.23.

Bauer M, Pretorius HW, Constant EL, Earley WR, Szamosi J, et al. (2009) Extended-Release Quetiapine as Adjunct to an Antidepressant in Patients With Major Depressive Disorder: Results of a Randomized, Placebo-Controlled, Double-Blind Study. Journal of Clinical Psychiatry 70(4): 540-549.

Baumann P, Souche A, Montaldi S, Baettig D, Lambert S, et al. (1996) A Double-Blind, Placebo-Controlled Study of Citalopram With and Without Lithium in the Treatment of Therapy-Resistant Depressive Patients: A Clinical, Pharmacokinetic, and Pharmacogenetic Investigation. Journal of Clinical Psychopharmacology 16(4): 307-314.

Berman RM, Fava M, Thase ME, Trivedi MH, Swanink R, et al. (2009) Aripiprazole Augmentation in Major Depressive Disorder: A Double-Blind, Placebo-Controlled Study in Patients with Inadequate Response to Antidepressants. CNS Spectrums 14(4): 197-206.

Berman RM, Marcus RN, Swanink R, McQuade RD, Carson WH, et al. (2007) The efficacy and safety of aripiprazole as adjunctive therapy in major depressive disorder: a multicenter, randomized, double-blind, placebo-controlled study. Journal of Clinical Psychiatry 68: 843-853.

Chaput Y, Magnan A and Gendron A (2008) The co-administration of quetiapine or placebo to cognitive-behavior therapy in treatment refractory depression: A preliminary trial. *BMC Psychiatry* 8: 73.

Cheon EJ, Lee KH, Park YW, Lee JH, Koo BH, et al. (2017) Comparison of the Efficacy and Safety of Aripiprazole Versus Bupropion Augmentation in Patients With Major Depressive Disorder Unresponsive to Selective Serotonin Reuptake Inhibitors: A Randomized, Prospective, Open-Label Study. *Journal of Clinical Psychopharmacology* 37: 193-199.

Corya SA, Williamson D, Sanger TM, Briggs SD, Case M, et al. (2006) A Randomized, Double-Blind Comparison Of Olanzapine/Fluoxetine Combination, Olanzapine, Fluoxetine, And Venlafaxine In Treatment-Resistant Depression. *Depression and Anxiety* 23: 364–372.

Cusin C, Iovieno N, Iosifescu DV, Nierenberg AA, Fava M, et al. (2013) A Randomized, Double-Blind, Placebo-Controlled Trial of Pramipexole Augmentation in Treatment-Resistant Major Depressive Disorder. *Journal of Clinical Psychiatry* 74(7): e636-e641.

Daly EJ, Singh JB, Fedgchin M, Cooper K, Lim P, et al. (2018) Efficacy and Safety of Intranasal Esketamine Adjunctive to Oral Antidepressant Therapy in Treatment-Resistant Depression A Randomized Clinical Trial. *JAMA Psychiatry* 75(2): 139-148.

DeBattista C, Doghramji K, Menza MA, Rosenthal MH and Fieve RR (2003) Adjunct Modafinil for the Short-Term Treatment of Fatigue and Sleepiness in Patients With Major Depressive Disorder: A Preliminary Double-Blind, Placebo-Controlled Study. *Journal of Clinical Psychiatry* 64: 1057-1064.

Dorèe JP, Des Rosiers J, Lew V, Gendron A, Elie R, et al. (2007) Quetiapine augmentation of treatment-resistant depression: a comparison with lithium. *Current Medical Research and Opinion* 23(2): 333-341.

Dunner DL, Amsterdam JD, Shelton RC, Loebel A and Romano SJ (2007) Efficacy and Tolerability of Adjunctive Ziprasidone in Treatment-Resistant Depression: A Randomized, Open-Label, Pilot Study. *Journal of Clinical Psychiatry* 68: 1071-1077.

Durgam S, Earley W, Guo H, Li D, Nèmeth G, et al. (2017) Efficacy and Safety of Adjunctive Cariprazine in Inadequate Responders to Antidepressants: A Randomized, Double-Blind, Placebo-Controlled Study in Adult Patients With Major Depressive Disorder. *Journal of Clinical Psychiatry* 77(3): 371-378.

Earley WR, Guo H, Németh G, Harsányi J and Thase ME (2018) Cariprazine augmentation to antidepressant therapy in major depressive disorder: results of a randomized, double-blind, placebo-controlled trial. *Psychopharmacology bulletin*, 48(4), p.62.

Eisendrath SJ, Gillung E, Delucchi KL, Segal ZV, Nelson C, et al. (2016) A Randomized Controlled Trial of Mindfulness-Based Cognitive Therapy for Treatment-Resistant Depression. *Psychotherapy and Psychosomatics* 85(2): 99-110.

El-Khalili N, Joyce M, Atkinson S, Buynak RJ, Datto C, et al. (2010) Extended-release quetiapine fumarate (quetiapine XR) as adjunctive therapy in major depressive disorder (MDD) in patients with an inadequate response to ongoing antidepressant treatment: a multicentre, randomized, double-blind, placebo-controlled study. *International Journal of Neuropsychopharmacology* 13: 917-932.

Fang Y, Yuan C, Xu Y, Chen J, Wu Z, et al. (2011) A Pilot Study of the Efficacy and Safety of Paroxetine Augmented With Risperidone, Valproate, Buspirone, Trazodone, or Thyroid Hormone in Adult Chinese Patients With Treatment-Resistant Major Depression. *Journal of Clinical Psychopharmacology* 31(5): 638-642.

Fava M, Alpert J, Nierenberg A, Lagomasino I, Sonawalla S, et al. (2002) Double-Blind Study of High-Dose Fluoxetine Versus Lithium or Desipramine Augmentation of Fluoxetine in Partial Responders and Nonresponders to Fluoxetine. *Journal of Clinical Psychopharmacology* 22: 379-387.

Fava M, Durgam S, Earley W, Lu K, Hayes R, et al. (2018) Efficacy of adjunctive low-dose cariprazine in major depressive disorder: a randomized, double-blind, placebo-controlled trial. *International Clinical Psychopharmacology* 33: 312-321.

Fava M, Memisoglu A, Thase ME, Bodkin JA, Trivedi MH, et al. (2016) Opioid Modulation With Buprenorphine/Samidorphan as Adjunctive Treatment for Inadequate Response to Antidepressants: A Randomized Double-Blind Placebo-Controlled Trial. *American Journal of Psychiatry* 173: 499-508.

Fava M, Mischoulon D, Iosifescu D, Witte J, Pencina M, et al. (2012) A Double-Blind, Placebo-Controlled Study of Aripiprazole Adjunctive to Antidepressant Therapy among Depressed Outpatients with Inadequate Response to Prior Antidepressant Therapy (ADAPT-A Study). *Psychotherapy and Psychosomatics* 81: 87-97.

Fava M, Rosenbaum JF, McGrath P, Stewart JW, Amsterdam JD, et al. (1994) Lithium and Tricyclic Augmentation of Fluoxetine Treatment for Resistant Major Depression: a Double-Blind Controlled Study. *American Journal of Psychiatry* 151: 1372-1374.

Fedgchin M, Trivedi M, Daly EJ, Melkote R, Lane R, et al. (2019) Efficacy and Safety of Fixed-Dose Esketamine Nasal Spray Combined With a New Oral Antidepressant in Treatment-Resistant Depression: Results of a Randomized, Double-Blind, Active-Controlled Study (TRANSFORM-1). *International Journal of Neuropsychopharmacology* 22(10): 616-630.

Fonagy P, Rost F, Carlyle J, McPherson S, Thomas R, et al. (2015) Pragmatic randomized controlled trial of long-term psychoanalytic psychotherapy for treatment-resistant depression: the Tavistock Adult Depression Study (TADS). *World Psychiatry* 14: 312-321.

Fornaro M, Martino M, Mattei C, Prestia D, Vinciguerra V, et al. (2014) Duloxetine-bupropion combination for treatment-resistant atypical depression: A double-blind, randomized, placebo-controlled trial. *European Neuropsychopharmacology* 24: 1269-1278.

Franco-Chaves JA, Mateus CF, Luckenbaugh DA, Martinez PE, Mallinger AG, et al. (2013) Combining a dopamine agonist and selective serotonin reuptake inhibitor for the treatment of depression: A double-blind, randomized pilot study. *Journal of Affective Disorders* 149(0): 319-325.

Gamble JJ, Bi H, Bowen R, Weisgerber G, Sanjanwala R, et al. (2018) Ketamine-based anesthesia improves electroconvulsive therapy outcomes: a randomized-controlled study. *Canadian Journal of Anesthesia* 65: 636-646.

George TP, Sacco KA, Vessicchio JC, Weinberger AH and Shytle RD (2008) Nicotinic Antagonist Augmentation of Selective Serotonin Reuptake Inhibitor–Refractory Major Depressive Disorder A Preliminary Study. *Journal of Clinical Psychopharmacology* 28: 340-344.

Girlanda F, Cipriani A, Agrimi E, Appino MG, Barichello A, et al. (2014) Effectiveness of lithium in subjects with treatment-resistant depression and suicide risk: results and lessons of an underpowered randomised clinical trial. *BMC Research Notes* 7: 731.

Gitlin MJ, Weiner H, Fairbanks L, Hershman JM, Friedfeld N. Failure of T3 to potentiate tricyclic antidepressant response. *Journal of affective disorders.* 1987 Nov 1;13(3):267-72.

Gulrez G, Badyal DK, Deswal RS, Sharma A. Bupropion as an augmenting agent in patients of depression with partial response. Basic & clinical pharmacology & toxicology. 2012 Mar;110(3):227-30.

Han C, Wang SM, Kwak KP, Won WY, Lee H, et al. (2015) Aripiprazole augmentation versus antidepressant switching for patients with major depressive disorder: A 6-week, randomized, rater-blinded, prospective study. *Journal of Psychiatric Research* 66-67: 84-94.

Harley R, Sprich S, Safren S, Jacobo M and Fava M (2008) Adaptation of Dialectical Behavior Therapy Skills Training Group for Treatment-Resistant Depression. *The Journal of Nervous and Mental Disease* 196: 136-143.

Hauksson P, Ingibergsdóttir S, Gunnarsdóttir T and Jónsdóttir IH (2017) Effectiveness of cognitive behaviour therapy for treatment-resistant depression with psychiatric comorbidity: comparison of individual versus group CBT in an interdisciplinary rehabilitation setting. *Nordic Journal Of Psychiatry* 71(6): 465-472.

Heresco-Levy U, Gelfin G, Bloch B, Levin R, Edelman S, et al. (2013) A randomized add-on trial of high-dose D-cycloserine for treatment-resistant depression. *International Journal of Neuropsychopharmacology* 16: 501-506.

Heresco-Levy U, Javiit DC, Gelfin Y, Gorelik E, Bar M, et al. (2006) Controlled trial of D-cycloserine adjuvant therapy for treatment-resistant major depressive disorder. *Journal of Affective Disorders* 93: 239-243.

Hobart M, Skuban A, Zhang P, Josiassen MK, Hefting N, et al. (2018a) Efficacy and safety of flexibly dosed brexpiprazole for the adjunctive treatment of major depressive disorder: a randomized, active-referenced, placebo- controlled study. Current Medical Research And Opinion 34(4): 633-642.

Hobart M, Skuban A, Zhang P, Augustine C, Brewer C, et al. (2018b) A Randomized, Placebo-Controlled Study of the Efficacy and Safety of Fixed-Dose Brexpiprazole 2 mg/d as Adjunctive Treatment of Adults With Major Depressive Disorder. *Journal of Clinical Psychiatry* 79(4): 17m12058.

# Horikoshi S, Miura I, Ichinose M, Yamamoto S, Ito M, et al. (2019) Low‐ and high‐dose aripiprazole augmentation and plasma levels of homovanillic acid in major depressive disorder: A randomized, open‐label study. *Human Psychopharmacology Clinical and Experimental* 34: e2696.

# Husain MI, Chaudhry IB, Husain N, Khoso AB, Rahman RR, et al. (2017) Minocycline as an adjunct for treatment- resistant depressive symptoms: A pilot randomised placebo-controlled trial. *Journal of Psychopharmacology* 31(9): 1166-1175.

Ionescu DF, Bentley KH, Eikermann M, Taylor N, Akeju O, et al. (2019) Repeat-dose ketamine augmentation for treatment-resistant depression with chronic suicidal ideation: A randomized, double blind, placebo controlled trial. *Journal of Affective Disorders* 243: 516-524.

Ionescu D, Dehelean C, Funar-Timofei S, Dragan S and Galca E (2010) Efficacy And Tolerability Of Risperidone In The Treatment Of Depressive Disorders. *Farmacia* 58(4): 494-501.

Joffe RT and Singer W (1990) A Comparison of Triiodothyronine and Thyroxine in the Potentiation of Tricyclic Antidepressants. *Psychiatry Research* 32: 241-251.

# Joffe RT, Singer W, Levitt AJ and MacDonald C (1993) A placebo-controlled comparison of lithium and triiodothyronine augmentation of tricyclic antidepressants in unipolar refractory depression. *Archives of General Psychiatry* 50(5): 387-393.

# Joffe RT, Sokolov S and Levitt AJ (2006) Lithium and Triiodothyronine Augmentation of Antidepressants. *Canadian Journal of Psychiatry* 51(12): 791-793.

# Kamijima K, Higuchi T, Ishigooka J, Ohmori T, Ozaki N, et al. (2013) Aripiprazole augmentation to antidepressant therapy in Japanese patients with major depressive disorder: A randomized, double-blind, placebo-controlled study (ADMIRE study). *Journal of Affective Disorders* 151: 899-905.

Kamijima K, Kimura M, Kuwahara K, Kitayama Y and Tadori Y (2018) Randomized, double-blind comparison of aripiprazole/sertraline combination and placebo/sertraline combination in patients with major depressive disorder. *Psychiatry and Clinical Neurosciences* 72: 591-601.

Keitner GI, Garlow SJ, Ryan CE, Ninan PT, Solomon DA, Nemeroff CB and Keller MB (2009) A randomized, placebo-controlled trial of risperidone augmentation for patients with difficult-to-treat unipolar, non-psychotic major depression. Journal of psychiatric research. 43(3):205-14.

Kessler DS, MacNeill SJ, Tallon D, Lewis G, Peters TJ, et al. (2018) Mirtazapine added to SSRIs or SNRIs for treatment resistant depression in primary care: phase III randomised placebo controlled trial (MIR). *BMJ* 363: k4218.

Kocsis JH, Gelenberg AJ, Rothbaum BO, Klein DN, Trivedi MH, et al. Cognitive Behavioral Analysis System of Psychotherapy and Brief Supportive Psychotherapy for Augmentation of Antidepressant Nonresponse in Chronic Depression: The REVAMP Trial. *Archives of General Psychiatry* 66(11): 1178-1188.

Kok RM, Vink D, Heeren TJ and Nolen WA (2007) Lithium Augmentation Compared With Phenelzine in Treatment-Resistant Depression in the Elderly: An Open, Randomized, Controlled Trial. *Journal of Clinical Psychiatry* 68: 1177-1185.

Lenze EJ, Mulsant BH, Blumberger DM, Karp JF, Newcomer JW, et al. (2015) Efficacy, safety, and tolerability of augmentation pharmacotherapy with aripiprazole for treatment-resistant depression in late life: a randomized placebo-controlled trial. *Lancet* 386: 2404-2412.

Lin C, Karim HT, Pecina M, Aizenstein HJ, Lenze EJ, et al. (2019) Low-dose augmentation with buprenorphine increases emotional reactivity but not reward activity in treatment resistant mid- and late-life depression. *NeuroImage: Clinical* 21: 101679.

Lin CH, Yang, WC, Chen CC and Cai WR (2020) Comparison of the efficacy of ECT plus agomelatine to ECT plus placebo in treatment-resistant depression. *Acta Psychiatrica Scandinavica* 142(2): 121-131.

# Maes M, Vandoolaeghe E and Desnyder R (1996) Efficacy of treatment with trazodone in combination with pindolol or fluoxetine in major depression. *Journal of Affective Disorders* 41: 201-210.

# Mahmoud RA, Pandina GJ, Turkoz I, Kosik-Gonzalez C, Canuso CM, et al. (2007) Risperidone for Treatment-Refractory Major Depressive Disorder A Randomized Trial. *Annals of Internal Medicine* 147: 593-602.

Mantani A, Kato T, Furukawa TA, Horikoshi M, Imai H, et al. (2017) Smartphone Cognitive Behavioral Therapy as an Adjunct to Pharmacotherapy for Refractory Depression: Randomized Controlled Trial. *Journal of Medical Internet Research* 19(11): e373.

Marcus RN, McQuade RD, Carson WH, Hennicken D, Fava M, et al. (2008) The Efficacy and Safety of Aripiprazole as Adjunctive Therapy in Major Depressive Disorder: A Second Multicenter, Randomized, Double-Blind, Placebo-Controlled Study. *Journal of Clinical Psychopharmacology* 28: 156-165.

Mathew SJ, Gueorguieva R, Brandt C, Fava M and Sanacora G (2017) A Randomized, Double-Blind, Placebo-Controlled, Sequential Parallel Comparison Design Trial of Adjunctive Riluzole for Treatment-Resistant Major Depressive Disorder. *Neuropsychopharmacology* 42: 2567-2574.

McAllister-Williams RH, Anderson IM, Finkelmeyer A, Gallagher P, Grunze HCR, et al. (2016) Antidepressant augmentation with metyrapone for treatment-resistant depression (the ADD study): a double-blind, randomised, placebo-controlled trial. *Lancet Psychiatry* 3: 117-127.

McGrath PJ, Stewart JW, Fava M, Trivedi MH, Wisniewski SR, et al. (2006) Tranylcypromine Versus Venlafaxine Plus Mirtazapine Following Three Failed Antidepressant Medication Trials for Depression: A STAR*D Report. *American Journal of Psychiatry* 163: 1531-1541.

McIntyre A, Gendron A and McIntyre A (2007) Quetiapine Adjunct To Selective Serotonin Reuptake Inhibitors Or Venlafaxine In Patients With Major Depression, Comorbid Anxiety, And Residual Depressive Symptoms: A Randomized, Placebo-Controlled Pilot Study. *Depression and Anxiety* 24: 487-494.

Medhus A, Heskestad S and Tjemsland L (1994) Mianserin added to tricyclic antidepressants in depressed patients not responding to a tricyclic antidepressant alone: A randomized, placebo- controlled, double-blind study. *Nordic Journal of Psychiatry* 48(5): 355-358.

Mohamed S, Johnson GR, Chen P, Hicks PB, Davis LL, et al. (2017) Effect of Antidepressant Switching vs Augmentation on Remission Among Patients With Major Depressive Disorder Unresponsive to Antidepressant Treatment The VAST-D Randomized Clinical Trial. *JAMA* 318(2): 132-145.

Möller HJ, Demyttenaere K, Olausson B, Szamosi J, Wilson E, et al. (2015) Two Phase III randomised double-blind studies of fixed-dose TC-5214 (dexmecamylamine) adjunct to ongoing antidepressant therapy in patients with major depressive disorder and an inadequate response to prior antidepressant therapy. *The World Journal of Biological Psychiatry* 16(7): 483-501.

Nakagawa A, Mitsuda D, Sado M, Abe T, Fujisawa D, et al. (2017) Effectiveness of Supplementary Cognitive-Behavioral Therapy for Pharmacotherapy-Resistant Depression: A Randomized Controlled Trial. *Journal of Clinical Psychiatry* 78(8): 1126-1135.

Nakao S, Nakagawa A, Oguchi Y, Mitsuda D, Kato N, et al. (2018) Web-Based Cognitive Behavioral Therapy Blended With Face-to-Face Sessions for Major Depression: Randomized Controlled Trial. *Journal Of Medical Internet Research* 20(9): e10743.

Navarro V, Boulahfa I, Obach A, Jerez D, Diaz-Ricart M, et al. (2019) Lithium Augmentation Versus Citalopram Combination in Imipramine-Resistant Major Depression A 10-Week Randomized Open-Label Study. *Journal of Clinical Psychopharmacology* 39: 254-257.

Nierenberg AA, Fava M, Trivedi MH, Wisniewski SR, Thase ME, et al. (2006) A Comparison of Lithium and T3 Augmentation Following Two Failed Medication Treatments for Depression: A STAR*D Report. *American Journal of Psychiatry* 163: 1519-1530.

Nierenberg AA, Papakostas GI, Petersen T, Montoya HD, Worthington JJ, et al. (2003) Lithium Augmentation of Nortriptyline for Subjects Resistant to Multiple Antidepressants. *Journal of Clinical Psychopharmacology* 23: 92-95.

Ochs-Ross R, Daly EJ, Zhang Y, Lane R, Lim P, et al. (2020) Efficacy and Safety of Esketamine Nasal Spray Plus an Oral Antidepressant in Elderly Patients With Treatment-Resistant Depression—TRANSFORM-3. *American Journal of Geriatric Psychiatry* 28(2): 121-141.

Papakostas GI, Fava M, Baer L, Swee MB, Jaeger A, et al. (2015) Ziprasidone Augmentation of Escitalopram for Major Depressive Disorder: Efficacy Results From a Randomized, Double-Blind, Placebo-Controlled Study. *American Journal of Psychiatry* 172(12): 1251-1258.

Papakostas GI, Mischoulon D, Shyu I, Alpert JE and Fava M (2010) S-Adenosyl Methionine (SAMe) Augmentation of Serotonin Reuptake Inhibitors for Antidepressant Nonresponders With Major Depressive Disorder: A Double-Blind, Randomized Clinical Trial. *American Journal of Psychiatry* 167: 942-948.

Papakostas GI, Shelton RC, Zajecka JM, Etemad B, Rickels K, et al. (2012) L-Methylfolate as Adjunctive Therapy for SSRI-Resistant Major Depression: Results of Two Randomized, Double-Blind, Parallel-Sequential Trials. *American Journal of Psychiatry* 169: 1267-1274.

Patkar AA, Masand PS, Pae CU, Peindl K, Hooper-Wood C, et al. (2006) A Randomized, Double-blind, Placebo-controlled Trial of Augmentation With an Extended Release Formulation of Methylphenidate in Outpatients With Treatment-Resistant Depression. *Journal of Clinicalsychopharmacology* 26: 653-656.

Pèrez V, Soler J, Puigdemont D, Alvarez E and Artigas F (1999) A Double-blind, Randomized, Placebo-Controlled Trial of Pindolol Augmentation in Depressive Patients Resistant to Serotonin Reuptake Inhibitors. *Archives of General Psychiatry* 56: 375-379.

Perry EB, Berman RM, Sanacora G, Anand A, Lynch-Colonese K, et al. (2004) Pindolol Augmentation in Depressed Patients Resistant to Selective Serotonin Reuptake Inhibitors: A Double-Blind, Randomized, Controlled Trial. *Journal of Clinical Psychiatry* 65: 238-243.

Pope HG, Amiaz R, Brennan BP, Orr G, Wesier M, et al. (2010) Parallel-Group Placebo-Controlled Trial of Testosterone Gel in Men With Major Depressive Disorder Displaying an Incomplete Response to Standard Antidepressant Treatment. *Journal of Clinical Psychopharmacology* 30: 126-134.

Pope HG, Cohane GH, Kanayama G, Siegel AJ and Hudson JI (2003) Testosterone Gel Supplementation for Men With Refractory Depression: A Randomized, Placebo-Controlled Trial. *American Journal of Psychiatry* 160: 105-111.

Popova V, Daly EJ, Trivedi M, Cooper K, Lane R, et al. (2019) Efficacy and Safety of Flexibly Dosed Esketamine Nasal Spray Combined With a Newly Initiated Oral Antidepressant in Treatment-Resistant Depression: A Randomized Double-Blind Active-Controlled Study. *American Journal of Psychiatry* 176(6): 428-438.

Ravindran AV, Kennedy SH, O'Donovan C, Fallu A, Camacho F, et al. (2008) Osmotic-Release Oral System Methylphenidate Augmentation of Antidepressant Monotherapy in Major Depressive Disorder: Results of a Double-Blind, Randomized, Placebo-Controlled Trial. *Journal of Clinical Psychiatry* 69: 87-94.

Reeves H, Batra S, May RS, Zhang R, Dahl DC, et al. (2008) Efficacy of Risperidone Augmentation to Antidepressants in the Management of Suicidality in Major Depressive Disorder: A Randomized, Double-Blind, Placebo-Controlled Pilot Study. *Journal of Clinical Psychiatry* 69: 1228-1236.

Richards C, Iosifescu DV, Mago R, Sarkis E, Reynolds J, et al. (2017) A randomized, double-blind, placebo-controlled, dose-ranging study of lisdexamfetamine dimesylate augmentation for major depressive disorder in adults with inadequate response to antidepressant therapy. *Journal of Psychopharmacology* 31(9): 1190-1203.

Richards C, McIntyre RS, Weisler R, Sambunaris A, Brawman-Mintzer O, et al. (2016) Lisdexamfetamine dimesylate augmentation for adults with major depressive disorder and inadequate response to antidepressant monotherapy: Results from 2 phase 3, multicenter, randomized, double-blind, placebo-controlled studies. *Journal of Affective Disorders* 206: 151-160.

Santos MA, Rocha FL and Hara C (2008) Efficacy and Safety of Antidepressant Augmentation With Lamotrigine in Patients With Treatment-Resistant Depression: A Randomized, Placebo-Controlled, Double-Blind Study. *Primary Care Companion to the Journal of Clinical Psychiatry* 10: 187-190.

Schindler F and Anghelescu IG (2007) Lithium versus lamotrigine augmentation in treatment resistant unipolar depression: a randomized, open-label study. *International Clinical Psychopharmacology* 22: 179-182.

Seidman SN, Miyazaki M and Roose SP (2005) Intramuscular testosterone supplementation to selective serotonin reuptake inhibitor in treatment-resistant depressed men: randomized placebo-controlled clinical trial. *Journal of Clinical Psychopharmacology* 25(6): 584-588.

Shelton RC, Tollefson GD, Tohen M, Stahl S, Gannon KS, et al. (2001) A Novel Augmentation Strategy for Treating Resistant Major Depression. *American Journal of Psychiatry* 158: 131-134.

Shelton RC, Williamson DJ, Corya SA, Sanger TM, Van Campen LE, et al. (2005) Olanzapine/Fluoxetine Combination for Treatment-Resistant Depression: A Controlled Study of SSRI and Nortriptyline Resistance. *Journal of Clinical Psychiatry* 66: 1289-1297.

Singh JB, Fedgchin M, Daly EJ, De Boer P, Cooper K, et al. (2016) A Double-Blind, Randomized, Placebo-Controlled, Dose-Frequency Study of Intravenous Ketamine in Patients With Treatment-Resistant Depression. *American Journal of Psychiatry* 173: 816-826.

Souza LH, Salum GA, Mosqueiro BP, Caldieraro MA, Guerra TA, et al. (2016) Interpersonal psychotherapy as add-on for treatment-resistant depression: A pragmatic randomized controlled trial. *Journal of Affective Disorders* 193: 373-380.

Stabl M, Kasas A, Blajev B, Bajetta G, Zochling R, et al. (1995) A Double-Blind Comparison of Moclobemide and Thioridazine Versus Moclobemide and Placebo in the Treatment of Refractory, Severe Depression. *Journal of Clinical Psychopharmacology* 15(4): 41S-45S.

Su TP, Chen MH, Li CT, Lin WC, Hong CJ, et al. (2017) Dose-Related Effects of Adjunctive Ketamine in Taiwanese Patients with Treatment-Resistant Depression. *Neuropsychopharmacology* 42: 2482–2492.

Targum SD, Cameron BR, Ferreira L and MacDonald ID (2018) An augmentation study of MSI-195 (S-adenosylmethionine) in Major Depressive Disorder. *Journal of Psychiatric Research* 107: 86-96.

Thase ME, Corya SA, Osuntokun O, Case M, Henley DB, et al. (2007b) A Randomized, Double-Blind Comparison of Olanzapine/Fluoxetine Combination, Olanzapine, and Fluoxetine in Treatment-Resistant Major Depressive Disorder. *Journal of Clinical Psychiatry* 68: 224-236.

Thase ME, Friedman ES, Biggs MM, Wisniewski SR, Trivedi MH, et al. (2007a) Cognitive Therapy Versus Medication in Augmentation and Switch Strategies as Second-Step Treatments: A STAR*D Report. *American Journal of Psychiatry* 164: 739-752.

Thase ME, Youakim JM, Skuban A, Hobart M, Augustine C, et al. (2015a) Efficacy and Safety of Adjunctive Brexpiprazole 2 mg in Major Depressive Disorder: A Phase 3, Randomized, Placebo-Controlled Study in Patients With Inadequate Response to Antidepressants. *Journal of Clinical Psychiatry* 76(9): 1224-1231.

Thase ME, Youakim JM, Skuban A, Hobart M, Zhang P, et al. (2015b) Adjunctive Brexpiprazole 1 and 3 mg for Patients With Major Depressive Disorder Following Inadequate Response to Antidepressants: A Phase 3, Randomized, Double-Blind Study. *Journal of Clinical Psychiatry* 76(9): 1232-1240.

Town JM, Abbass A, Stride C and Bernier D (2017) A randomised controlled trial of Intensive Short-Term Dynamic Psychotherapy for treatment resistant depression: the Halifax Depression Study. *Journal of Affective Disorders* 214: 15-25.

Trivedi MH, Fava M, Wisniewski SR, Thase ME, Quitkin F, et al. (2006) Medication Augmentation after the Failure of SSRIs for Depression. *New England Journal of Medicine* 354: 1243-1252.

Wiles NJ, Hollinghurst S, Mason V and Musa M (2008) A Randomized Controlled Trial of Cognitive Behavioural Therapy as an Adjunct to Pharmacotherapy in Primary Care Based Patients with Treatment Resistant Depression: A Pilot Study. *Behavioural and Cognitive Psychotherapy* 36: 21-33.

Wiles NJ, Thomas L, Abel A, Barnes M, Carroll F, et al. (2014) Clinical effectiveness and cost-effectiveness of cognitive behavioural therapy as an adjunct to pharmacotherapy for treatment-resistant depression in primary care: the CoBalT randomised controlled trial. *Health Technology Assessment* 18: 31.

Wiles NJ, Thomas L, Abel A, Ridgway N, Turner N, et al. (2013) Cognitive behavioural therapy as an adjunct to pharmacotherapy for primary care based patients with treatment resistant depression: results of the CoBalT randomised controlled trial. *Lancet* 381: 375-384.

Yoshimura R, Hori H, Umene-Nakano W, Ikenouchi-Sugita A, Katsuki A, et al. (2014) Comparison of lithium, aripiprazole and olanzapine as augmentation to paroxetine for inpatients with major depressive disorder. *Therapeutic Advances in Psychopharmacology* 4(3): 123-129.

Yoshimura R, Kishi T, Hori H, Ikenouchi-Sugita A, Katsuki A, et al. (2012) Comparison of the efficacy between paroxetine and sertraline augmented with aripiprazole in patients with refractory major depressive disorder. *Progress in Neuro-Psychopharmacology & Biological Psychiatry* 39: 355-357.

Zajecka JM, Stanford AD, Memisoglu A, Martin WF and Pathak S (2019) Buprenorphine/samidorphan combination for the adjunctive treatment of major depressive disorder: results of a phase iii clinical trial (FORWARD-3). *Neuropsychiatric Disease and Treatment* 15: 795-808.

Zusky PM, Biederman J, Rosenbaum JF, Manschreck TC, Gross CC, et al. (1988) Adjunct Low Dose Lithium Carbonate in Treatment-Resistant Depression: A Placebo-Controlled Study. *Journal of Clinical Psychopharmacology* 8(2): 120-124.
